# Genetic diversity of *Xanthoceras sorbifolium* bunge germplasm using morphological traits and microsatellite molecular markers

**DOI:** 10.1371/journal.pone.0177577

**Published:** 2017-06-01

**Authors:** Zhan Shen, Jie Duan, Luyi Ma

**Affiliations:** 1 National Energy R&D Center for Non-food Biomass, Beijing Forestry University, Beijing, China; 2 Key Laboratory for Silviculture and Conservation, Ministry of Education, Beijing Forestry University, Beijing, China; University of Lagos, NIGERIA

## Abstract

*Xanthoceras sorbifolium* Bunge has great potential for producing biodiesel. In order to select and evaluate appropriate germplasm to produce biodiesel, we analyzed the genetic diversity of *Xanthoceras sorbifolium* Bunge germplasm based on morphological traits and simple sequence repeats (SSRs) in this study. Fifty-six germplasm samples were evaluated using nine morphological traits and 23 SSR loci. Significant differences among germplasms were observed in eight morphological characters. The SSR markers analysis showed high genetic diversity among the germplasms. All SSRs had polymorphisms, and we detected 77 alleles in total. The number of alleles at each locus ranged from two to six, averaging 3.35 per marker. The polymorphic information content values ranged from 0.36 to 0.61, averaging 0.49. Expected heterozygosity, observed heterozygosity, and Shannon’s information index calculations detected large genetic variations among germplasms. The high average number of alleles per locus and the allelic diversity observed in the set of genotypes analyzed indicated that the genetic base of this species is relatively wide. Thus, microsatellite markers can be used to efficiently distinguish *Xanthoceras sorbifolium* Bunge germplasms and assess their genetic diversity. Hundred-grain weight and lateral diameter were positively correlated with monounsaturated fatty acids and depended on genotype. These results suggest that seeds with higher hundred-grain weight and lateral diameter could be more suitable to produce biodiesel. Our data will lay a foundation for selecting appropriate germplasm to produce biodiesel based on seed phenotype and will contribute to the conservation and management of this important plant genetic resource.

## Introduction

*Xanthoceras sorbifolium* Bunge (Family Sapindaceae) is a small tree that produces edible fruit and seeds with high oil content. The plants are long lived (up to 1000 years) and tolerant of drought, low temperature, alkaline soils, and low fertility. Because of high monounsaturated fatty acid contents, the oil from this plant has considerable potential for producing biodiesel [1,2]. However, due to rapid expansion in production, planting of unknown cultivars and use of low-quality planting material has often occurred. In addition, the number of similar or related cultivars is growing rapidly because of limited parental germplasm resources for breeding, which can cause varietal complexity in the seedling market. Mixed seedlings and cultivars result in many different seed shapes and have had a negative impact on seed processing.

Simple sequence repeats (SSRs) reflect genetic diversity due to their high levels of allelic variation and their codominant character, which allows them to deliver more information per unit assay than other marker systems [3]. SSR markers have also been used widely to understand genetics and the relationships between many species, such as *Jatropha curcas* L. [4], *Vitis vinifera* L. [5,6], *Liriodendron chinense* (Hemsl.) Sarg. [7,8], *Juglans* [9], and *Olea europaea* L. [10]. Several types of molecular markers have been used to discriminate cultivars and to study the genetic diversity and relationships of *Xanthoceras sorbifolium* Bunge, such as random amplification of DNA ends (RAPD) [11] and intersimple sequence repeat (ISSR) [12,13]. Although a few SSR markers have been developed for *Xanthoceras sorbifolium* Bunge [14], the utility of SSR primers to identify varieties and perform a genetic diversity analysis of *Xanthoceras sorbifolium* Bunge germplasm has not been determined.

The objectives of this study were to provide valuable seed phenotype and fatty acid content information to select the appropriate cultivar for producing biodiesel and identify 56 mixed seedling and cultivar germplasms using SSR markers and morphological traits. We analyzed the relationship between seed phenotype and monounsaturated fatty acid content to determine which characters were positively correlated with monounsaturated fatty acids. The Mantel test was performed between the selected characters and genotypes to understand whether the selected characters depended on genotype. These will be useful as a reference for biodiesel production from *Xanthoceras sorbifolium* Bunge, as well as for rapidly and effectively screening *Xanthoceras sorbifolium* Bunge germplasm based on seed phenotype.

## Material and methods

### Plant material and seed phenotype

Seeds and young leaf tissues of 56 mixed seedling and cultivar germplasms were collected from Ongniud Banner, Inner Mongolia, China (119.10°E, 42.37°N). Seed phenotype characters were evaluated, including 100-grain weight, transverse diameter, longitudinal diameter, and lateral diameter. Each sample was analyzed three times. Data are reported as the mean ± standard deviation (SD).

### Kernel oil content

Seeds were dried to constant weight at 80°C and then pulverized in a ball mill. The kernel oil components were extracted with petroleum ether (boiling point, 60°C) using a Soxhlet extraction device (Soxtec 8000; FOSS, Hillerød, Denmark). The extraction process included boiling at 120°C for 5 min, leaching for 1 h, and recovery for 25 min. Kernel oil weight was calculated by the weight difference between the sample extracts. Each sample was analyzed three times. Data are reported as the mean ± SD.

### Transesterification experiments and methyl ester analysis

For each sample, 0.06 g of oil was placed in a 10-mL capped test tube with a mixture of 4 mL isooctane and 0.2 mL potassium hydroxide-methanol (2 M) following the GB/T 17376–2008 method. After the oil sample was dissolved, the solution was vortexed for 30 s. Then, 1 g of sodium bisulfate was added to neutralize excess alkalinity, followed by 15 s of vortexing. After clarification, the supernatant was transferred to a vial for analysis by gas chromatography-mass spectrometry (GC-MS) to determine biodiesel yield and fatty acid composition.

The fatty acid methyl ester (FAME) composition of the seed kernel oil was measured with GC-MS using an HP-INNOWAX capillary column (30 m × 0.25 mm × 0.25 μm, model 7890A; Agilent Technologies, Santa Clara, CA, USA). The column temperature was held at 160°C for 1 min, heated to 250°C at 4°C/min, and held constant for 5 min. Nitrogen was used as the carrier gas at a flow rate of 25 mL/min. The injector and detector temperatures were set to 220 and 275°C, respectively. The hydrogen and air flow rates were set to 30 and 400 mL/min, respectively. FAME content was quantified by comparison with an external standard (37 component FAME Mix, purity, 97.8–99.9%; Supelco, Bellefonte, PA, USA). The fatty acid qualitative analysis was performed using the standard peak retention times of fatty acids and the MS library, and the quantitative analysis was conducted by measuring peak area.

### DNA extraction

Genomic DNA was extracted from young leaf tissues of each germplasm using the Takara MiniBEST Plant Genomic DNA Extraction Kit (Dalian, China). After 0.8% agarose gel electrophoresis, DNA concentration was quantified using a NanoDrop ND-1000 spectrophotometer (NanoDrop Technologies, Wilmington, DE, USA). The DNA concentration was adjusted to 20 ng/μL, and the quality of the product was evaluated by determining that the 260/280 nm and 260/230 nm absorbance ratios were ≥ 1.8 [15].

### SSR markers and polymerase chain reaction (PCR) amplification

Thirty-eight genomic highly polymorphic SSR markers developed by Bi and Guan (2014) [14] were used to assess the genetic diversity in 56 samples. PCR amplifications of all primers were performed in a total volume of 10 μL containing 20 ng DNA, 0.2 μM forward primer, 0.2 μM reverse primer, and 5 μL RR901A mix (Takara Bio). PCR amplifications were performed in a thermal cycler (T100; Bio-Rad, Hercules, CA, USA) using the following sequence: initial step at 94°C for 3 min, followed by 30 cycles of denaturation at 94°C for 30 s, annealing at 55°C for 30 s, and extension at 72°C for 1 min. The final 10-min extension was performed at 72°C. The PCR products were checked by 1.5% agarose gel electrophoresis. Subsequently, the primers with corresponding bands were resolved by non-denaturing polyacrylamide gel electrophoresis and visualized by silver nitrate staining to check the DNA banding patterns. Polymorphic bands were used for the identification step. Reproducibility of the PCR procedures was confirmed by repeating the process three times.

### Data analysis

Seed phenotype, kernel oil contents, and FAME composition were examined by analysis of variance (ANOVA). The distance matrices were based on the Gower general similarity coefficient [16]. Cluster analyses were performed using the unweighted pair group method with arithmetic mean (UPGMA) procedure and NTSYS-pc 2.11 software (Exeter Software, Stauket, NY, USA).

The polymorphic bands in the SSR marker analysis were scored as either present (1) or absent (0). The alleles were coded alphabetically (e.g., A, B, or C for three bands) in order of decreasing size. The number of alleles per locus (Na), the number of effective alleles per locus (Ne), the Shannon index, observed heterozygosity (Ho), and expected heterozygosity (He) were calculated using POPGENE 32 software [17]. Polymorphism information content (PIC) was estimated using Power Stats V12.xls software [18]. A matrix of genetic distances [19] was constructed for the 56 germplasms. A dendrogram cluster analysis was performed with NTSYS-pc 2.11 software using the UPGMA procedure [20]. The Mantel test was performed to examine the relationships between morphological characters and genetic distance among the 56 germplasms.

## Results

### Seed phenotype and oil characteristics

Seed phenotype and oil characteristics for the 56 *Xanthoceras sorbifolium* Bunge germplasms were determined (Table 1 and S1 Table). Among the 56 germplasms, mean 100-grain weight, transverse diameter, longitudinal diameter, lateral diameter, kernel percentage, kernel oil content, saturated fatty acids, monounsaturated fatty acids, and polyunsaturated fatty acids were 90.41 ± 15.68g, 1.29 ± 0.11cm, 1.41 ± 0.12cm, 1.06 ± 0.11cm, 54.81 ± 5.48%, 54.12 ± 3.72%, 8.33 ± 0.41%, 43.81 ± 1.75%, and 47.69 ± 2.01%, respectively. Each character was compared among the 56 germplasms using ANOVA. No differences in transverse diameter were observed, but highly significant differences (P < 0.01) were detected for the other eight characters among the 56 germplasms.

**Table 1:** Seed phenotype and oil characteristics of the 56 *Xanthoceras sorbifolium* Bunge germplasms.

No.	Hundred-grain weight (g) ^**^	Transverse diameter (cm)	Longitudinal diameter (cm) ^**^	Lateral diameter (cm) ^**^	Kernel percentage (%)^**^	Kernel oil content (%)^**^	Saturated fatty acids (%)^**^	Monounsaturated fatty acids (%)^**^		Polyunsaturated fatty acids (%)^**^
1	111.54 ± 0.16	1.41 ± 0.16	1.45 ± 0.18	1.14 ± 0.04	58.40 ± 0.26	51.84 ± 0.03	8.13 ± 0.42		43.02 ± 0.02	48.86 ± 0.45
2	108.35 ± 0.21	1.22 ± 0.09	1.58 ± 0.18	1.08 ± 0.02	63.71 ± 0.16	54.49 ± 0.53	8.43 ± 0.02	47.64 ± 0.10		43.79 ± 0.11
3	86.89 ± 0.05	1.16 ± 0.20	1.32 ± 0.10	0.94 ± 0.05	53.36 ± 0.11	50.94 ± 0.08	7.89 ± 0	42.09 ± 0.04		49.80 ± 0.04
4	89.56 ± 0.13	1.39 ± 0.04	1.56 ± 0.08	1.04 ± 0.03	56.27 ± 0.24	52.60 ± 0.15	7.96 ± 0.05	42.21 ± 0.23		49.67 ± 0.30
5	79.64 ± 0.08	1.32 ± 0.04	1.41 ± 0.27	0.98 ± 0.04	62.12 ± 0.88	53.46 ± 0.62	8.06 ± 0.03	42.68 ± 0.07		49.05 ± 0.04
6	88.41 ± 0.20	1.38 ± 0.03	1.51 ± 0.09	1.20 ± 0.11	56.60 ± 0.45	49.10 ± 0.09	8.25 ± 0.04	42.34 ± 0.04		49.21 ± 0
7	113.59 ± 0.44	1.39 ± 0.16	1.63 ± 0.04	1.12 ± 0.05	53.26 ± 0.32	57.63 ± 0.34	8.62 ± 0.03	45.66 ± 0.12		45.62 ± 0.06
8	72.14 ± 0.34	1.24 ± 0.12	1.29 ± 0.13	1.12 ± 0.17	56.29 ± 0.25	51.86 ± 0.06	7.83 ± 0.04	41.72 ± 0.20		50.29 ± 0.25
9	102.49 ± 0.01	1.31 ± 0.10	1.38 ± 0.11	0.97 ± 0.13	51.44 ± 0.45	50.74 ± 0.23	8.57 ± 0.04	43.98 ± 0.07		47.32 ± 0.02
10	66.38 ± 0.11	1.22 ± 0.03	1.38 ± 0.06	0.88 ± 0.06	53.54 ± 0.48	55.23 ± 0.20	7.44 ± 0.03	41.81 ± 0.04		50.66 ± 0.07
11	50.69 ± 0.30	0.98 ± 0.10	1.08 ± 0.05	0.81 ± 0.13	47.72 ± 0.25	57.56 ± 0.59	8.62 ± 0.04	42.90 ± 0.18		48.37 ± 0.20
12	72.25 ± 0.21	1.25 ± 0.15	1.24 ± 0.03	1.04 ± 0.06	50.40 ± 0.13	56.88 ± 0.08	8.61 ± 0.06	47.72 ± 0.02		43.59 ± 0.08
13	78.37 ± 0.23	1.13 ± 0.06	1.20 ± 0.08	1.00 ± 0.04	52.51 ± 0.25	53.60 ± 0.11	8.31 ± 0	42.60 ± 0.11		48.95 ± 0.11
14	96.37 ± 0.30	1.26 ± 0.17	1.37 ± 0.26	1.08 ± 0.08	52.92 ± 0.33	57.72 ± 0.25	8.57 ± 0.03	44.14 ± 0		47.04 ± 0.04
15	95.24 ± 0.06	1.31 ± 0.05	1.39 ± 0.17	1.14 ± 0.13	47.64 ± 0.37	51.51 ± 0.46	8.47 ± 0.25	45.26 ± 0		46.28 ± 0.25
16	84.80 ± 0.14	1.23 ± 0.20	1.40 ± 0.01	0.96 ± 0.08	56.44 ± 0.33	57.30 ± 0.27	7.89 ± 0.04	43.02 ± 0.13		48.99 ± 0.13
17	89.61 ± 0.37	1.37 ± 0.08	1.48 ± 0.05	1.08 ± 0.04	56.86 ± 0.05	59.43 ± 0.27	8.87 ± 0.53	47.46 ± 0.1		43.68±0.43
18	101.71 ± 0.21	1.40 ± 0.09	1.54 ± 0.03	1.09 ± 0.04	56.21 ± 0.07	59.19 ± 0.23	8.50 ± 0.08	46.67 ± 0.13		44.70 ± 0.04
19	90.49 ± 0.30	1.26 ± 0.01	1.42 ± 0.10	0.87 ± 0.13	54.22 ± 0.04	52.70 ± 0.06	8.25 ± 0.04	42.78 ± 0.18		48.86 ± 0.16
20	70.28 ± 0.25	1.13 ± 0.13	1.23 ± 0.04	0.94 ± 0.16	51.80 ± 0.25	56.72 ± 0.17	7.79 ± 0.03	42.11 ± 0.04		49.92 ± 0.07
21	77.14 ± 0.13	1.12 ± 0.29	1.28 ± 0.32	0.92 ± 0.02	61.02 ± 0.37	50.41 ± 0.16	7.92 ± 0.01	43.51 ± 0.14		48.38 ± 0.16
22	107.25 ± 0.07	1.40 ± 0.13	1.59 ± 0.04	1.07 ± 0.03	52.24 ± 0.63	53.67 ± 0.06	7.87 ± 0.01	41.45 ± 0.13		50.42 ± 0.11
23	82.71 ± 0.21	1.27 ± 0.13	1.33 ± 0.11	0.95 ± 0.04	57.76 ± 0.66	50.72 ± 0.09	8.23 ± 0.32	43.17 ± 0.21		48.60 ± 0.11
24	85.59 ± 0.44	1.38 ± 0.24	1.30 ± 0.01	1.16 ± 0.02	50.23 ± 0.24	54.54 ± 0.28	8.43±0.48	47.42 ± 0.04		43.51 ± 0.24
25	89.44 ± 0.09	1.24 ± 0.21	1.33 ± 0.20	1.00 ± 0.14	48.66 ± 0.87	61.20 ± 0.02	8.40 ± 0.25	45.05 ± 0.25		46.36 ± 0.60
26	80.24 ± 0.33	1.14 ± 0.08	1.31 ± 0.08	1.07 ± 0.10	46.42 ± 0.47	57.53 ± 0.03	8.17 ± 0	43.51 ± 0.01		48.22 ± 0.01
27	93.37±0.11	1.19 ± 0.06	1.48 ± 0.03	0.98 ± 0.23	53.56 ± 0.37	52.32 ± 0.08	8.12 ± 0.03	42.16 ± 0.04		49.56 ± 0.06
28	97.46 ± 0.50	1.37 ± 0.06	1.50 ± 0.08	1.10 ± 0.04	51.26 ± 0.33	49.30 ± 0.26	8.23 ± 0.05	43.52 ± 0.02		48.14 ± 0
29	113.54 ± 0.08	1.41 ± 0.11	1.36 ± 0.15	1.28 ± 0.10	56.45 ± 0.35	55.38 ± 0.4	8.63 ± 0.01	43.20 ± 0.01		48.07 ± 0.03
30	73.27 ± 0.02	1.20 ± 0.06	1.42 ± 0.07	1.12 ± 0.03	57.37 ± 0.35	54.31 ± 0.04	8.37 ± 0.20	43.47 ± 0.49		47.91 ± 0.69
31	74.39 ± 0.52	1.26 ± 0.02	1.31 ± 0.18	0.87 ± 0.25	57.29 ± 0.04	53.01 ± 0	8.12 ± 0.01	42.48 ± 0.05		49.26 ± 0.03
32	89.66 ± 0.27	1.22 ± 0.10	1.34 ± 0.02	1.08 ± 0.03	51.65 ± 0.30	65.25 ± 0.33	8.50 ± 0	45.58 ± 0.04		45.80 ± 0.01
33	88.65 ± 0.35	1.31 ± 0.06	1.39 ± 0.02	1.06 ± 0.21	54.83 ± 0.55	52.44 ± 0.45	9.06 ± 0.06	44.67 ± 0.01		46.12 ± 0.07
34	91.25 ± 0.28	1.24 ± 0.08	1.53 ± 0.05	1.23 ± 0.01	62.83 ± 0.28	55.58 ± 0.12	8.39 ± 0.03	44.30 ± 0.10		47.05 ± 0.13
35	116.61 ± 0.34	1.53 ± 0.04	1.59 ± 0.01	1.38 ± 0.04	51.38 ± 0.28	53.58 ± 0.45	8.68 ± 0.07	45.30 ± 0		45.87 ± 0.07
36	87.61 ± 0.34	1.18 ± 0.13	1.34 ± 0.06	1.02 ± 0.07	50.95 ± 1.29	59.52 ± 0.61	8.06 ± 0.1	44.40 ± 0.32		47.42 ± 0.37
37	99.05 ± 0.01	1.35 ± 0.18	1.35 ± 0.10	1.15 ± 0.18	60.45 ± 0.49	57.50 ± 0.11	8.93 ± 0.03	44.20 ± 0.03		46.70 ± 0.01
38	66.66 ± 0.37	1.22 ± 0.08	1.37 ± 0.05	1.06 ± 0.04	56.50 ± 0.67	54.67 ± 0.01	8.40 ± 0	40.66 ± 0.01		50.80 ± 0.01
39	117.39 ± 0.02	1.46 ± 0.06	1.50 ± 0.05	1.21 ± 0.04	56.83 ± 0.13	58.48 ± 0.06	8.49 ± 0.38	45.35 ± 0.18		45.92 ± 0.57
40	109.37 ± 0.12	1.38 ± 0.11	1.47 ± 0.12	1.13 ± 0.06	48.52 ± 0.39	47.27 ± 0.08	9.54 ± 0.03	45.22 ± 0.14		45.04 ± 0.18
41	93.96 ± 0.01	1.28 ± 0.11	1.19 ± 0.07	1.05 ± 0.01	58.27 ± 0.18	54.41 ± 0.46	8.29 ± 0.03	42.28 ± 0.03		49.17 ± 0.01
42	80.65 ± 0.36	1.22 ± 0.11	1.43 ± 0.19	1.10 ± 0.11	50.33 ± 0.20	56.51 ± 0.01	7.61 ± 0.05	42.76 ± 0.04		49.43 ± 0.01
43	92.28 ± 0.04	1.31 ± 0.09	1.40 ± 0.06	1.01 ± 0.12	54.31 ± 0.16	51.53 ± 0.13	7.64 ± 0.03	43.74 ± 0.11		48.34 ± 0.13
44	77.35 ± 0.21	1.23 ± 0.01	1.51 ± 0.08	1.02 ± 0.04	62.24 ± 0.30	49.93 ± 0.11	8.33 ± 0.03	40.64 ± 0.16		50.87 ± 0.17
45	77.49 ± 0.16	1.16 ± 0.12	1.53 ± 0.16	1.01 ± 0.23	46.49 ± 0.61	47.36 ± 0.08	8.23 ± 0.03	44.06 ± 0.03		47.62 ± 0.06
46	91.34 ± 0.34	1.35 ± 0.06	1.46 ± 0.06	1.14 ± 0.06	52.49 ± 0.64	51.73 ± 0.21	8.29 ± 0.27	42.85 ± 0.01		48.70 ± 0.23
47	73.65 ± 0.36	1.19 ± 0.13	1.39 ± 0.05	1.04 ± 0.04	67.79 ± 0.58	50.81 ± 0.11	8.13 ± 0.04	43.19 ± 0.09		48.56 ± 0.06
48	107.49 ± 0.05	1.39 ± 0.03	1.39 ± 0.07	1.16 ± 0.02	56.51 ± 0.37	55.64 ± 0.04	8.42 ± 0.55	47.47 ± 0.21		43.94 ± 0.74
49	99.59 ± 0.45	1.39 ± 0.13	1.51 ± 0.06	1.23 ± 0.08	67.28 ± 0.56	55.62 ± 0.43	8.53 ± 0.09	46.48 ± 0.15		44.85 ± 0.23
50	79.41 ± 0.23	1.18 ± 0.16	1.32 ± 0.01	1.04 ± 0.01	47.62 ± 0.38	48.58 ± 0.34	8.19 ± 0.01	45.40 ± 0.02		46.21 ± 0.01
51	80.72 ± 0.37	1.35 ± 0.23	1.43 ± 0.11	0.98 ± 0.07	65.43 ± 0.29	53.29 ± 0.20	8.99 ± 0.64	46.81 ± 0.18		44.12 ± 0.71
52	88.54 ± 0.16	1.40 ± 0.14	1.39 ± 0.01	1.05 ± 0.16	47.60 ± 0.52	49.54 ± 0.39	7.95 ± 0.03	42.35 ± 0.03		49.54 ± 0.07
53	116.24 ± 0.30	1.36 ± 0.16	1.66 ± 0.06	1.13 ± 0.16	51.64 ± 0.23	52.68 ± 0.20	8.13 ± 0.06	42.31 ± 0.02		49.41 ± 0.01
54	78.43 ± 0.10	1.25 ± 0.18	1.31 ± 0.02	0.96 ± 0.11	42.21 ± 0.23	48.49 ± 0.67	8.08 ± 0.02	41.77 ± 0.02		49.99 ± 0
55	103.31 ± 0.22	1.28 ± 0.25	1.36 ± 0.12	1.04 ± 0.02	60.63 ± 0.36	52.13 ± 0.01	8.53 ± 0.06	45.84 ± 0.05		45.43 ± 0.11
56	133.32 ± 0.45	1.52 ± 0.11	1.46 ± 0.01	1.22 ± 0.02	60.65 ± 0.14	53.13 ± 0.06	8.51 ± 0	45.16 ± 0.11		46.09 ± 0.11
Total	90.41 ± 15.68	1.29 ± 0.11	1.41 ± 0.12	1.06 ± 0.11	54.81 ± 5.48	54.12 ± 3.72	8.33 ± 0.41	43.81 ± 1.75		47.69 ± 2.01

^**^Significant at the 1% level.

Data are reported as the mean ± standard deviation (SD).

**Fig 1:**
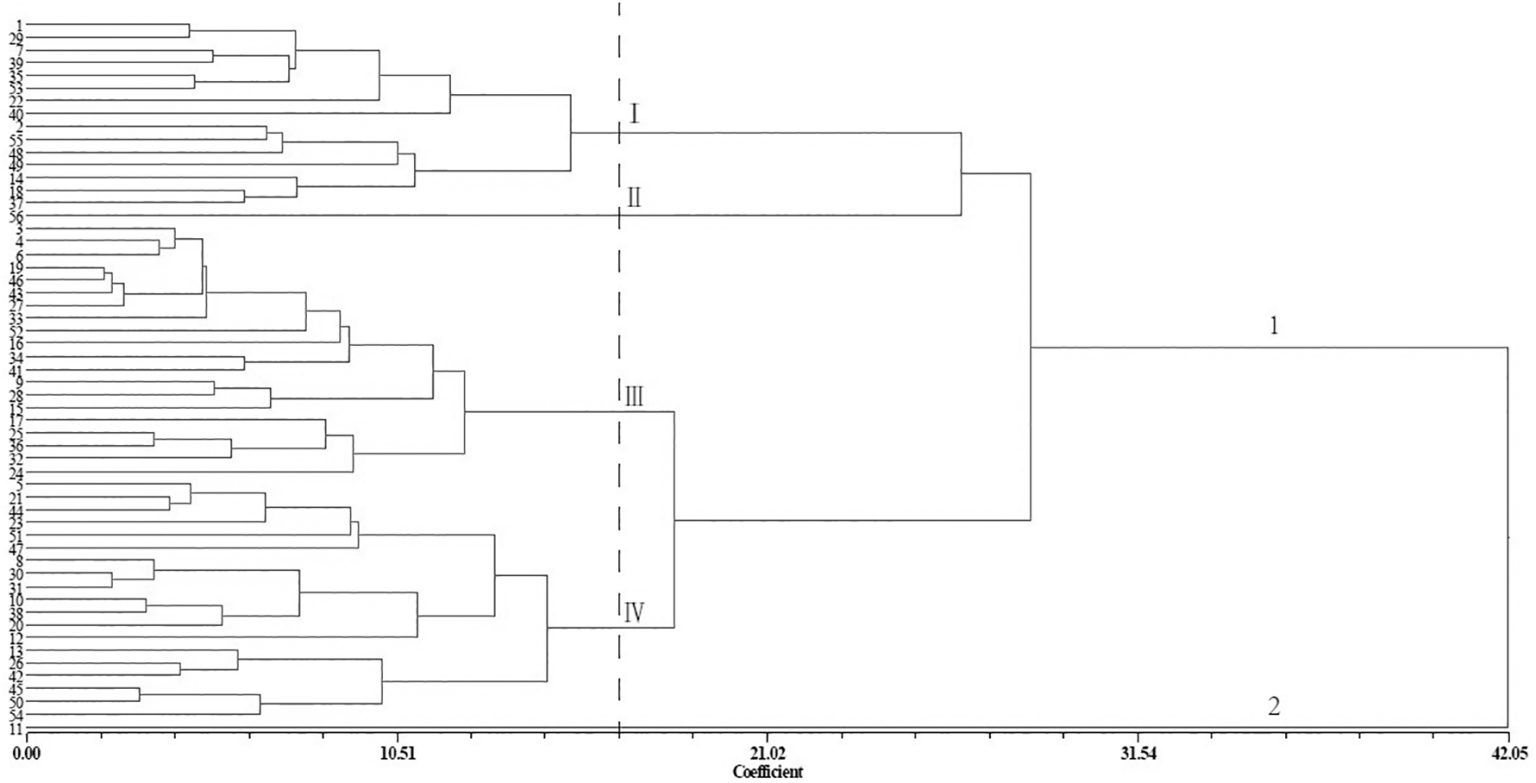
Agglomerative hierarchical cluster analysis of seed phenotype and oil characteristics of 56 *Xanthoceras sorbifolium* Bunge germplasms using the unweighted pair group method with arithmetic mean (UPGMA) procedure. Gower general similarity coefficients (Coefficient) were used to calculate genetic distances among the germplasms.

The nine characters were used to evaluate genetic distances among the germplasms and to construct a dendrogram (Fig 1). The 56 germplasms were classified into two main groups according to the nine characters. The second group contained only the no. 11 germplasm, which had the lowest 100-grain weight, longitudinal diameter, and lateral diameter. The first group was divided into four subgroups at a coefficient of 16.82. The first subgroup comprised 15 germplasms, which had higher 100-grain weight, kernel oil content, and monounsaturated fatty acids. The second subgroup included only the no. 56 germplasm, which had the highest 100-grain weight, transverse diameter, longitudinal diameter, and lateral diameter, as well as higher kernel percentage, kernel oil content, and monounsaturated fatty acids. The third subgroup contained 20 germplasms and average levels of these characters. The last subgroup contained the remaining 19 germplasms, which had lower 100-grain weight, kernel oil content, and monounsaturated fatty acids.

### Relationship between seed phenotype and oil characteristics

A partial correlation analysis was conducted to study the relationship between seed phenotype and oil characteristics (Table 2). The results showed that 100-grain weight was positively correlated with saturated fatty acids (r = 0.317^*^) and monounsaturated fatty acids (r = 0.348^**^), but negatively correlated with polyunsaturated fatty acids (r = −0.367^**^). Lateral diameter was positively correlated with saturated fatty acids (r = 0.336^*^) and monounsaturated fatty acids (r = 0.339^**^), but negatively correlated with polyunsaturated fatty acids (r = −0.367^**^).

**Table 2:** Partial correlation analysis between seed phenotype and oil characteristics of the 56 *Xanthoceras sorbifolium* Bunge germplasms.

	Kernel percentage	Kernel oil content	Saturated fatty acids	Monounsaturated fatty acids	Polyunsaturated fatty acids
Hundred-grain weight	0.13	0.063	0.317^*^	0.348^**^	−0.367^**^
Longitudinal diameter	0.214	−0.112	0.081	0.124	−0.121
Lateral diameter	0.147	0.131	0.336^**^	0.339^**^	−0.367^**^

^*^ Significant at the 5% level

^**^ Significant at the 1% level.

### SSR characterization

Considerable variation was observed in the amplified fragment patterns using different primers. Of the 38 primers tested, 15 yielded no amplification products; these are not included in our report. The remaining 23 SSR markers (Table 3) were used for the characterization and genetic diversity analyses of the 56 *Xanthoceras sorbifolium* Bunge germplasms (Table 4). And the electropherogram were showed in S1 Folder. Seventy-seven alleles were detected in total. The number of alleles (Na) values ranged from two (QXH002) to six (QXH274), averaging 3.35 alleles/locus across the 23 loci. All loci were polymorphic. Polymorphism information content (PIC) values ranged from 0.36 (QXH002 and QXH197) to 0.61 (QXH323), averaging 0.49. The mean expected heterozygosity (He) value was 0.58; values ranged from 0.45 in QBRS192 to 0.68 in QXH323. Observed heterozygosity (Ho) ranged from 21% in QXRB116 to 96% in QXH274, averaging 0.74. Wright’s fixation index (Fst) compares He and Ho, and is a measure of the degree of allelic fixation; Fst values ranged from 0.11 (QBLB62) to 0.82 (QXRB116), averaging 0.35. The Shannon-Weaver information index (I) ranged from 0.66 in QXH197 to 1.21 in QXH274 and QXH323, averaging 0.95. Thus, abundant genetic diversity were detected among the 56 germplasms. The most polymorphic locus, QXH323, had a high level of genetic variation. The high level of heterozygosity (average Ho = 0.74) detected by our SSR marker analysis indicated a high level of cross-pollination in *Xanthoceras sorbifolium* Bunge.

**Table 3:** Characterization of 23 microsatellite loci in *Xanthoceras sorbifolium* Bunge.

Locus	Repeat motifs	Primer sequences (5′–3′)	
QXH002	TC	F	AGAAGAACACTCAATGGGGA
		R	CTTCAACTGGACACCCGTAT
QXH049	CT	F	CCCCAACAAATGGTAAGACG
		R	GAATTTACAAGACAAGCAACAGC
QXH083	CT	F	AGCGGTCTCCTCCACTATCA
		R	GAATTGAAGCGCAGAAGGA
QXH120	AG	F	AAAACACTTCCGCACCAA
		R	TGGCTGCTGAGAAAGTAAGG
QXH177	AG	F	TGTGGTGGTTTTGGCAGAC
		R	CACCAAATAATGTCAATATCCTGT
QXH197	AG	F	GAAATATGAGGTCTTGGGTGTT
		R	GTGGCAGATAAACTGTCCTCAA
QXH262	CT	F	TCTAACCGAAGAAGCCAACT
		R	AGCGTGATATTCTGTTTCAACTAC
QXH274	CT	F	CATCGTCGTCTCATCCAGTAA
		R	GTGGCTTGTAGTTTGTTTCGTT
QXH282	CT	F	CCCAACAAATGGTAAGACGT
		R	GTTTCATTTCATTTCCAGCATC
QXH323	AG	F	CACAACCCAAATCCCAGAAC
		R	AACGACACGCACAATCATAAC
QXH365	AC	F	GTATATCTCTTTTACGGTCGTGAC
		R	ATGATGGGTTGGGTTGAGTT
QXHS371	GT	F	ATTGGAGTGGCCTTCATACG
		R	GCAAGCAGCTAAAGAAACAGC
QXH643	CA, CT	F	GCAGTTATGGAAAGGAATCA
		R	ATCAGTGTCGATTATTATCT
QXR343	TG	F	CACACTTTCTGAGTCCCGTAT
		R	TGTTTCTCCTCTAATCCAAC
QXR634	TTTTA, GA	F	CGTCCATCGCTTTCACCT
		R	AATAACAAATCAAAAATACCCCAT
QXR639	CT	F	CAACACCACCTCCACCAAC
		R	AAGGGATTTTGCTTTTCTGG
QXRB116	AC	F	CACCTTTCTCACTCCGTCTC
		R	CTCCTCGATCTGGTTGCTAA
QBRB203	AT	F	ACAAGTAGTGCTCATCGGTTTA
		R	GAGTCTAATAGGTAAGGCTAGGAAC
QBRS192	TC, CT	F	TTGTGCATCTGTGGAGAAGG
		R	TGCCTTCACATCCTGTGGTA
QBLB51	TC	F	TCCCACCCCAAAAAACTATAT
		R	CCTTCTGGAACTTGATGCC
QBLB58	TC, TA	F	CTCTTCCACTTCGGTCACG
		R	AACCAGAGCCACGCAAAT
QBLB62	CT	F	ATTCAGAAACGGTGGCTTAG
		R	TTATTTACTCTGGAAGGCTTATTT
QBLB65	TC	F	TATCTCGCCACATCTGCC
		R	CTGAAATGCCATTAACAAACAC

F, forward primer; R, reverse primer.

**Table 4:** Genetic diversity among the 56 *Xanthoceras sorbifolium* Bunge germplasms.

Locus	Na	Ne	I	Ho	He	Nei	Fst	PIC
QXH002	2	1.90	0.67	0.77	0.48	0.47	0.19	0.36
QXH049	4	2.36	1.04	0.80	0.58	0.58	0.30	0.52
QBLB51	3	2.57	1.02	0.95	0.62	0.61	0.22	0.54
QBLB58	3	2.22	0.86	0.89	0.55	0.55	0.19	0.45
QBLB62	3	2.09	0.79	0.93	0.53	0.52	0.11	0.41
QBLB65	3	2.51	0.98	0.77	0.61	0.60	0.36	0.52
QXH083	3	2.44	0.98	0.38	0.6	0.59	0.68	0.52
QXRB116	3	2.54	0.99	0.21	0.61	0.61	0.82	0.52
QXH120	3	2.27	0.92	0.75	0.56	0.56	0.33	0.48
QXH177	3	2.03	0.81	0.43	0.51	0.51	0.58	0.42
QBRS192	3	1.81	0.77	0.59	0.45	0.45	0.34	0.39
QXH197	2	1.87	0.66	0.52	0.47	0.46	0.44	0.36
QBRB203	3	2.27	0.90	0.93	0.56	0.56	0.17	0.47
QXH262	3	2.99	1.10	0.93	0.67	0.67	0.30	0.59
QXH274	6	2.80	1.21	0.96	0.65	0.64	0.25	0.58
QXH282	4	2.17	0.95	0.73	0.54	0.54	0.32	0.48
QXH323	4	3.03	1.21	0.91	0.68	0.67	0.32	0.61
QXR343	3	2.16	0.84	0.48	0.54	0.54	0.55	0.43
QXH365	3	2.73	1.05	0.93	0.64	0.63	0.27	0.56
QXHS371	3	2.77	1.06	0.93	0.64	0.64	0.27	0.57
QXR634	5	2.39	1.06	0.70	0.59	0.58	0.40	0.51
QXR639	5	2.51	1.12	0.64	0.61	0.60	0.47	0.55
QXH643	3	2.33	0.93	0.86	0.58	0.57	0.25	0.48
Mean	3.35	2.38	0.95	0.74	0.58	0.57	0.35	0.49
St. Dev	0.93	0.34	0.15	0.21	0.06	0.06	0.17	0.07

Na, number of alleles; Ne, number of effective alleles; Ho, observed heterozygosity; He, expected heterozygosity; I, Shannon’s diversity index; Nei, genetic diversity; Fst, fixation index; PIC, polymorphism information content.

### Cluster analyses

Because 100-grain weight and lateral diameter were positively correlated with monounsaturated fatty acids, it was considered whether 100-grain weight and lateral diameter were determined by genetics. Thus, a dendrogram cluster analysis and a Mantel test were performed.

**Fig 2:**
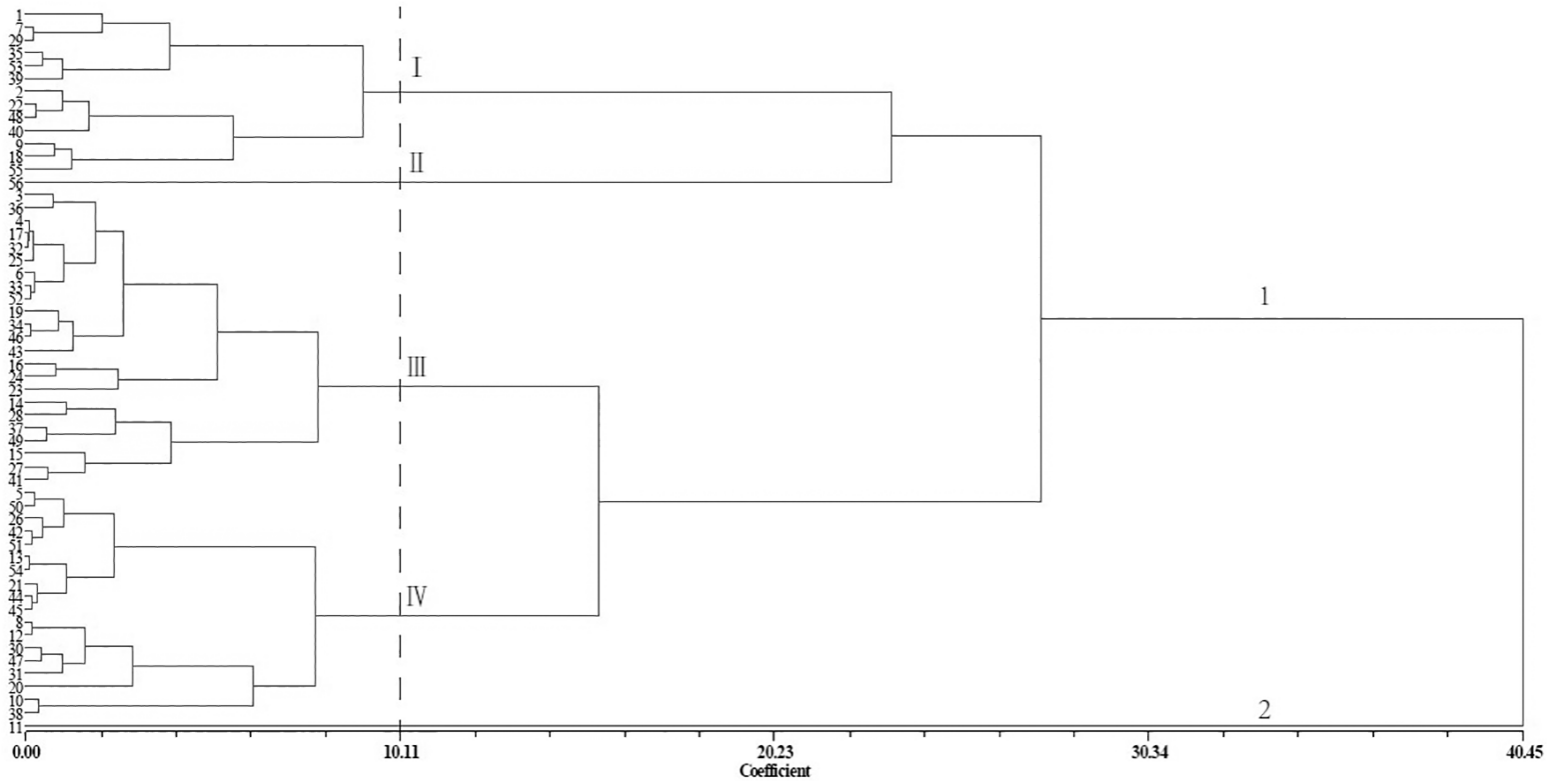
Agglomerative hierarchical cluster analysis of the hundred-grain weight and lateral diameter of 56 *Xanthoceras sorbifolium* Bunge germplasms using the UPGMA procedure. Gower general similarity coefficients (Coefficient) were used to calculate genetic distances among germplasms.

The 56 *Xanthoceras sorbifolium* Bunge germplasms clustered into two main groups based on 100-grain weight and lateral diameter. The second group contained only the no. 11 germplasm, which had the lowest 100-grain weight and lateral diameter. At a coefficient of 10.11, the first group was divided into four subgroups. The first subgroup contained 13 germplasms, which had higher 100-grain weight and lateral diameter values. The second subgroup included only the no. 56 germplasm, which had the highest 100-grain weight and lateral diameter values. The third subgroup comprised 23 germplasms, which had average 100-grain weight and lateral diameter values. The last subgroup contained the remaining 18 germplasms, which had lower 100-grain weight and lateral diameter values (Fig 2).

**Fig 3:**
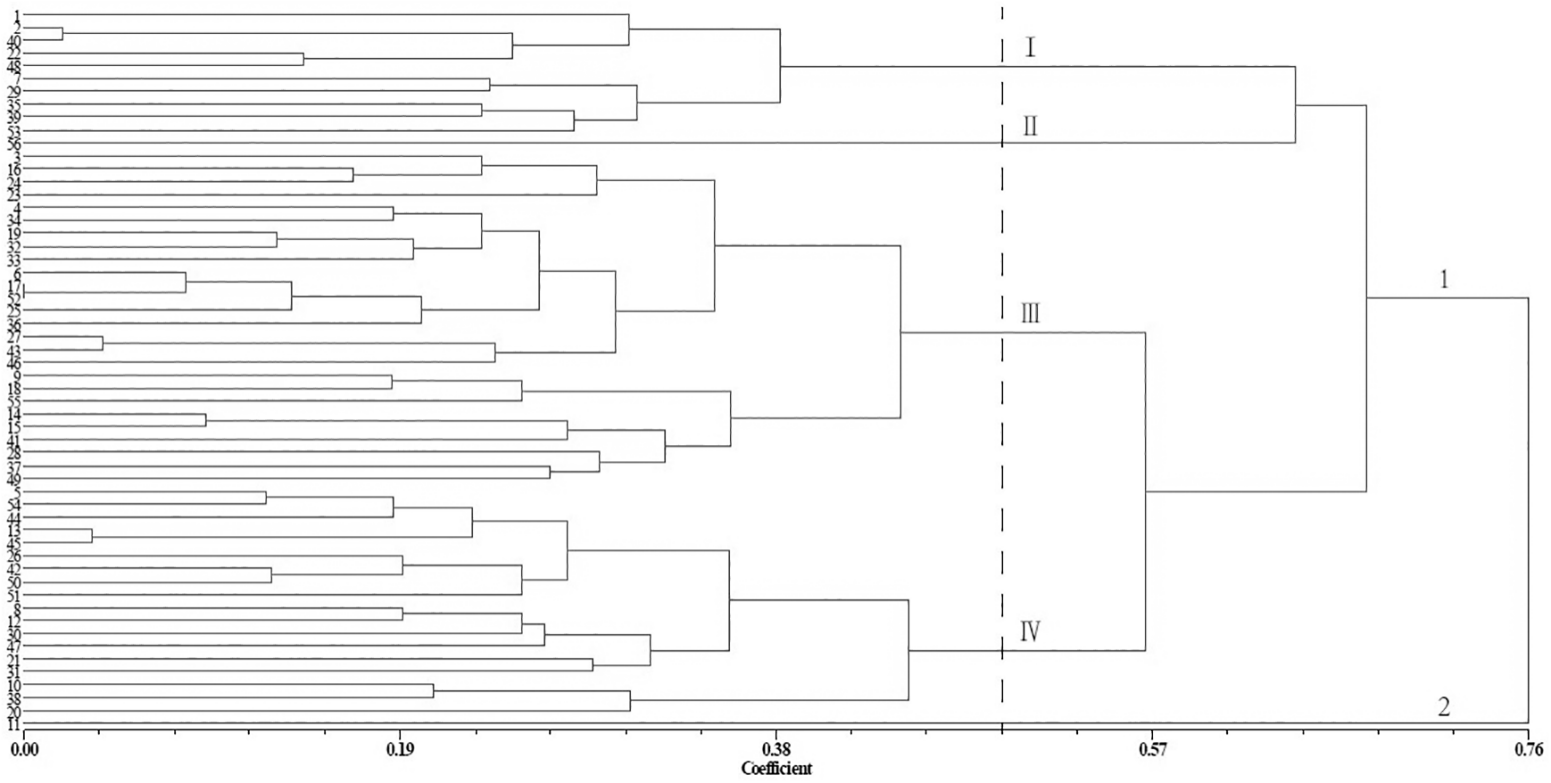
Agglomerative hierarchical cluster analysis of Nei’s pairwise genetic distances among 56 *Xanthoceras sorbifolium* Bunge germplasms using the UPGMA procedure. The dendrogram is based on analyses of 23 simple sequence repeat (SSR) loci.

Nei’s genetic distances was calculated to explore the genetic relationships among the 56 *Xanthoceras sorbifolium* Bunge germplasms. The genetic distance matrix was subjected to a UPGMA cluster analysis (Fig 3). The 56 germplasms were classified into two main groups, in which the second group contained only the no. 11 germplasm. At a coefficient of 0.50, the first group was divided into four subgroups. The first subgroup contained 10 germplasms. The second subgroup comprised only the no. 56 germplasm. The third subgroup comprised 26 germplasms. The last subgroup contained the remaining 18 germplasms.

**Fig 4:**
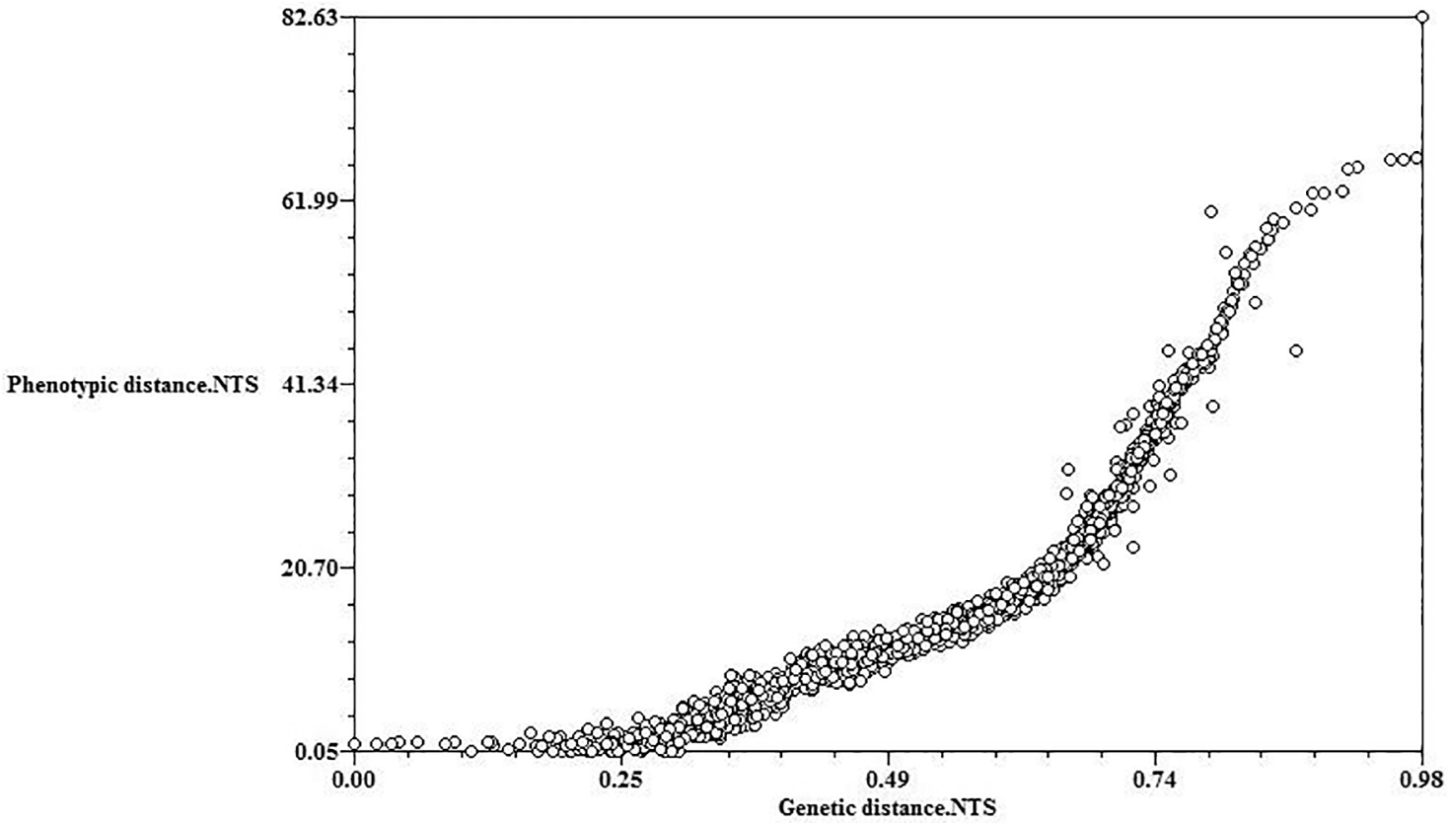
Mantel test analysis of the correlation between phenotypic and genetic distances in the 56 *Xanthoceras sorbifolium* Bunge germplasm.

The Mantel test results showed that the genetic and phenotypic distances of the 56 germplasms were significantly positively correlated (r = 0.92, P < 0.01) (Fig 4).

## Discussion

### Morphological variations

Variations in phenotypic traits are based on the variation and interactions at the genotypic level of the plant as well as the environmental pressure on the plant [21]. Although the 56 germplasms were in the same environment, they showed significant differences among the eight selected characters. This result illustrates that wild germplasm carries an important degree of genetic variation, which is vital for improving modern cultivars with domesticated and breeding-narrowed genetic backgrounds [22]. It was demonstrated here that seed quality includes important morphological traits, such as 100-grain weight, oil content, and monounsaturated fatty acids, among others, which can be used to improve the quality of biodiesel as reported previously [23]. Our results shows that seeds with higher 100-grain weight and lateral diameter values had higher monounsaturated fatty acid contents.

### SSR markers

Data on the relationships between genotypes help solve problems in breeding programs and germplasm resource management [24]. Many types of molecular markers, particularly SSR markers, have been used successfully to assess genetic diversity and characterize crop resources [25–27].

Molecular techniques based on DNA markers, such as RAPD and ISSR, have been used to characterize genetic diversity in *Xanthoceras sorbifolium* Bunge [11–13], but SSRs markers have not yet come into general use in studies for this species. An SSR analysis was used to investigate genetic diversity in 56 *Xanthoceras sorbifolium* Bunge germplasms, and 23 SSR polymorphic markers were highly informative. The proportion of polymorphic loci that obtained in this study (100.00%) was exceeded the proportions in previous RAPD and ISSR studies [11–13]. The mean number of alleles per locus was 3.35, and the average PIC value was 0.49. As demonstrated previously, the SSR assay approach is appropriate for studies of genetic relationships [10,28] and has proven to be an efficient tool for assessing genetic diversity of *Xanthoceras sorbifolium* Bunge and identifying its germplasm.

### Germplasm selection for producing biodiesel

Our Mantel test analysis detected a significantly positive correlation between phenotypic and genetic distances among the 56 germplasms (Fig 4), suggesting that these selected characters (100-grain weight and lateral diameter) depended on genotype. Because the two characters were positively correlated with monounsaturated fatty acids (Table 2), it was hypothesized that seeds with higher 100-grain weight and lateral diameter values would be more suitable for producing biodiesel. Among the 56 germplasms, no. 56 had the highest 100-grain weight and lateral diameter values. Thus, it was inferred that no. 56 would be the best germplasm to produce biodiesel.

Our results help understand the relationships between germplasm characters and genotype and will improve the *Xanthoceras sorbifolium* Bunge germplasm to achieve higher production of higher quality biodiesel. Our data will lay the foundation for selecting excellent germplasm to produce biodiesel based on seed phenotype, regardless of the environment.

## Conclusion

Our data showed significant variations in the morphological traits and microsatellite DNA polymorphisms among 56 *Xanthoceras sorbifolium* Bunge germplasms. The large average number of alleles per locus and allelic diversity in the set of genotypes analyzed indicate that the genetic spectrum was relatively wide. Our results show that SSR markers are a useful tool to explore *Xanthoceras sorbifolium* Bunge diversity. Hundred-grain weight and lateral diameter were positively correlated with monounsaturated fatty acids, and were dependent on genotype. These results suggest that seeds with higher 100-grain weight and lateral diameter values could be more suitable to produce biodiesel.

## Supporting information

S1 Table Seed phenotype and oil characteristics of the 56 *Xanthoceras sorbifolium* Bunge germplasms. This is the raw data of morphological traits.
(XLSX)

S1 Folder The electropherogram from the genetic diversity study. (ZIP)
