# Supplementary figures and images for: Genetic diversity of Xanthoceras sorbifolium bunge germplasm using morphological traits and microsatellite molecular markers
Source: PLoS One. 2017 Jun 1;12(6):e0177577. doi: 10.1371/journal.pone.0177577 (PMC5453433; doi:10.1371/journal.pone.0177577)

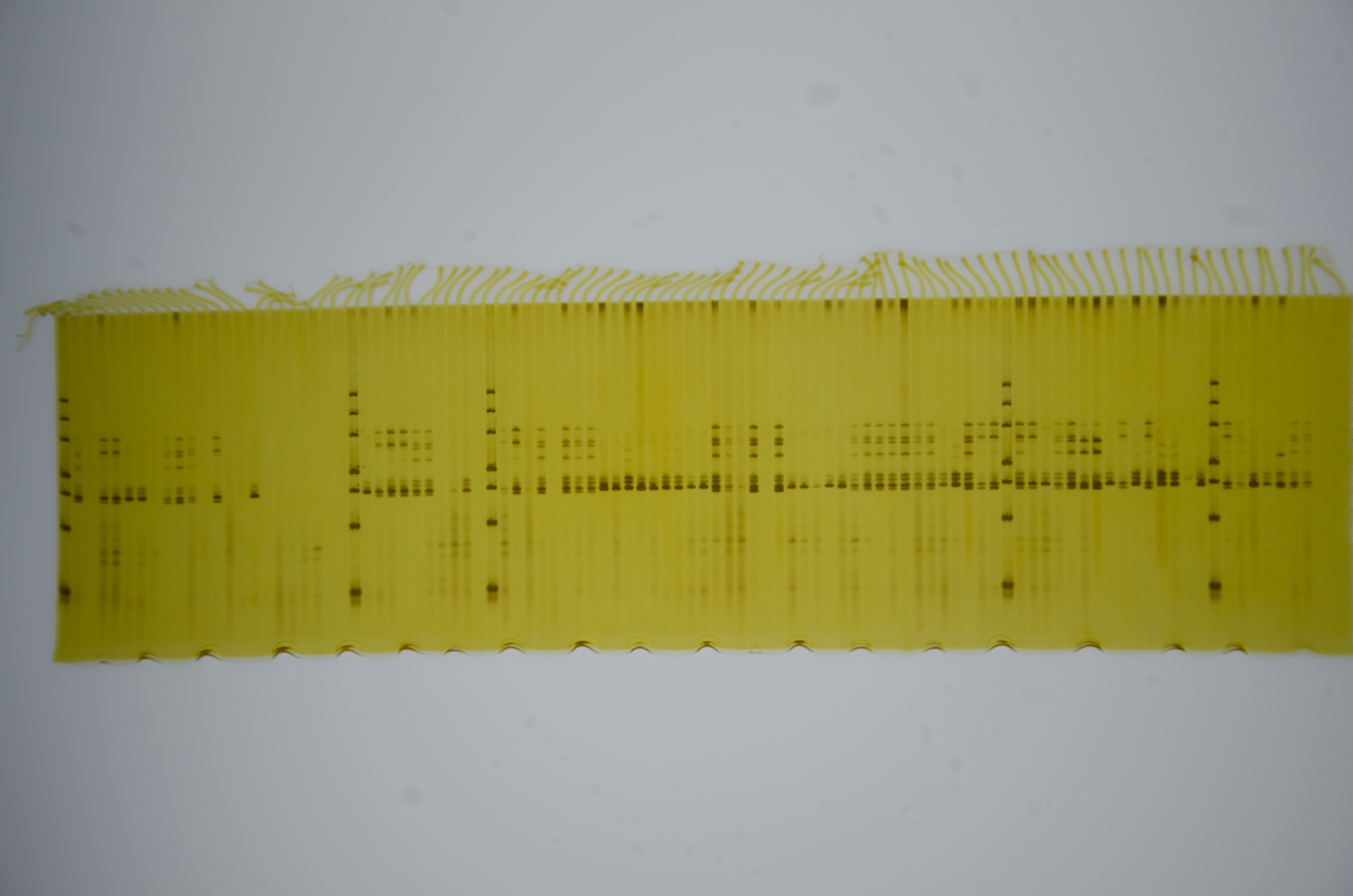

Supplement: S1 Folder — (ZIP) [file pone.0177577.s002.zip › S2 File/002.jpg]

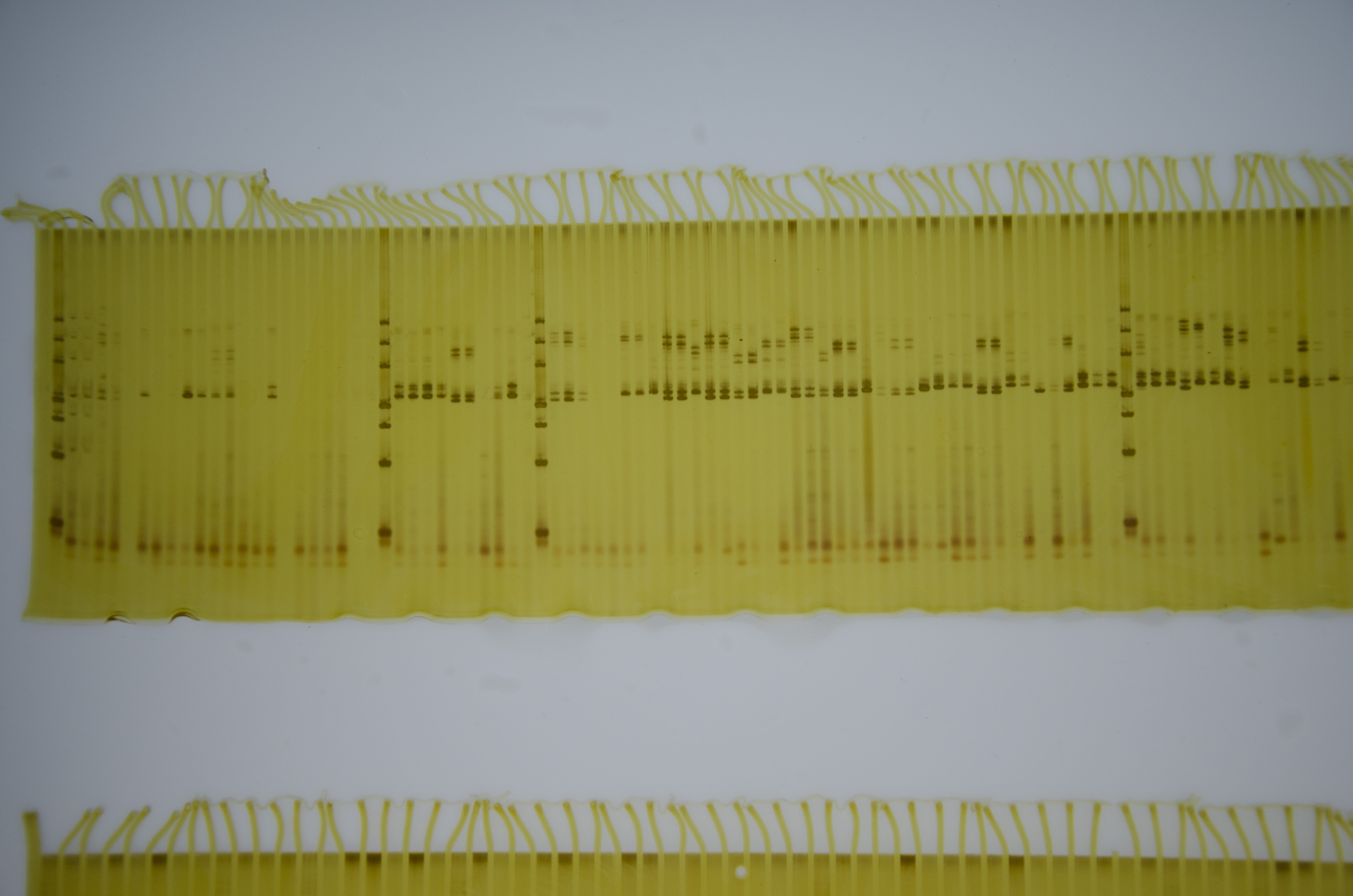

Supplement: S1 Folder — (ZIP) [file pone.0177577.s002.zip › S2 File/049.jpg]

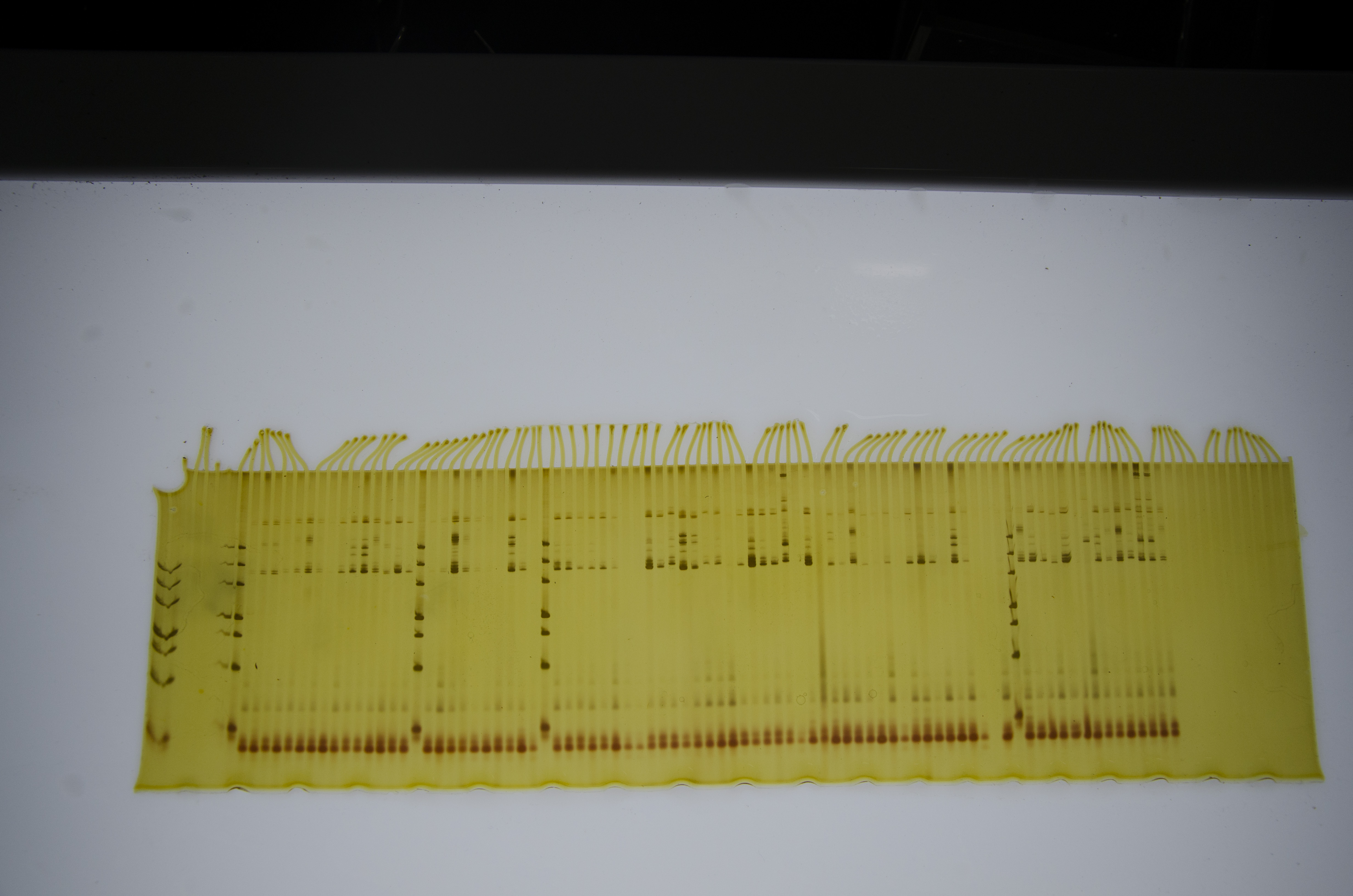

Supplement: S1 Folder — (ZIP) [file pone.0177577.s002.zip › S2 File/083.jpg]

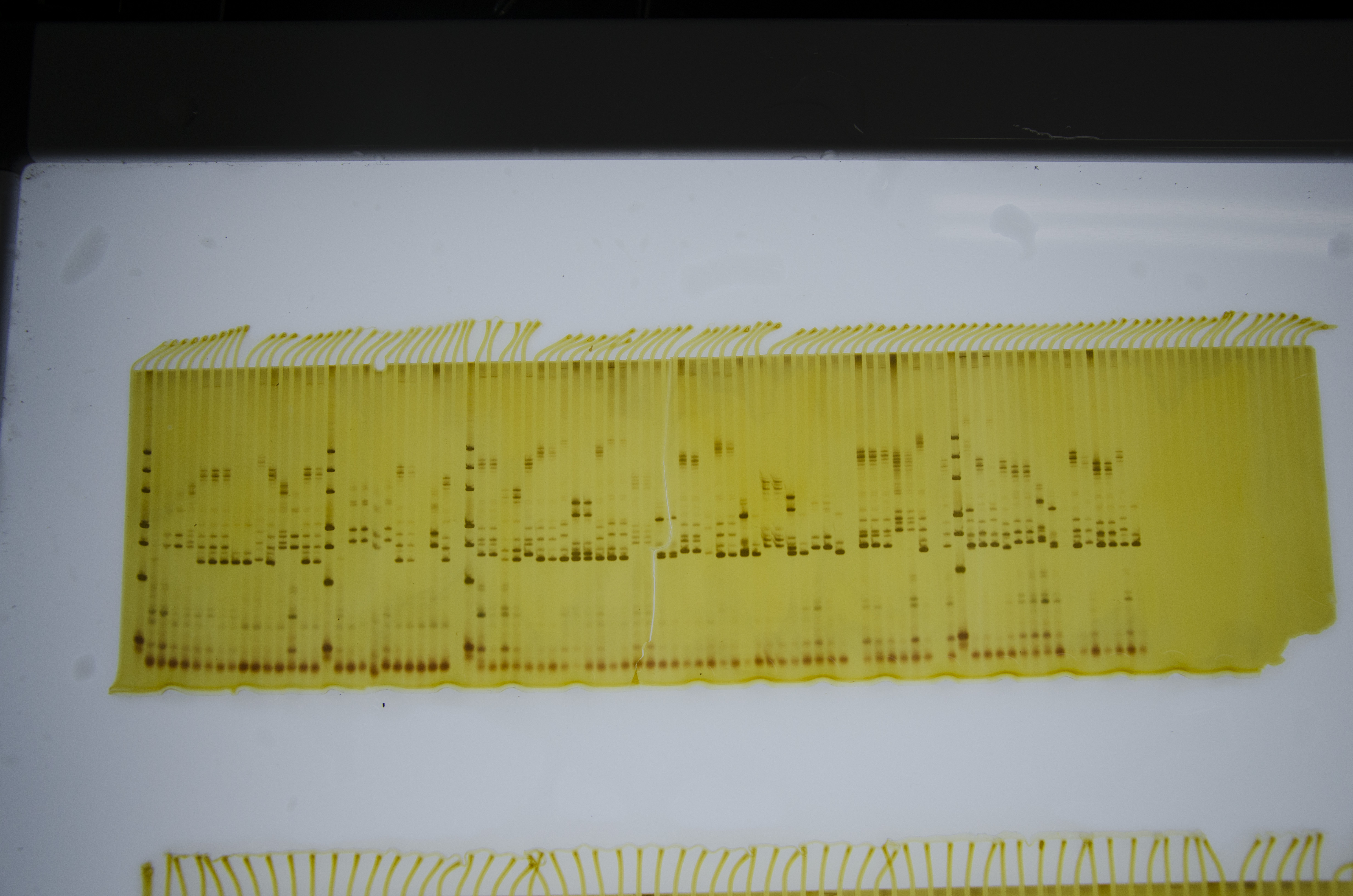

Supplement: S1 Folder — (ZIP) [file pone.0177577.s002.zip › S2 File/116.jpg]

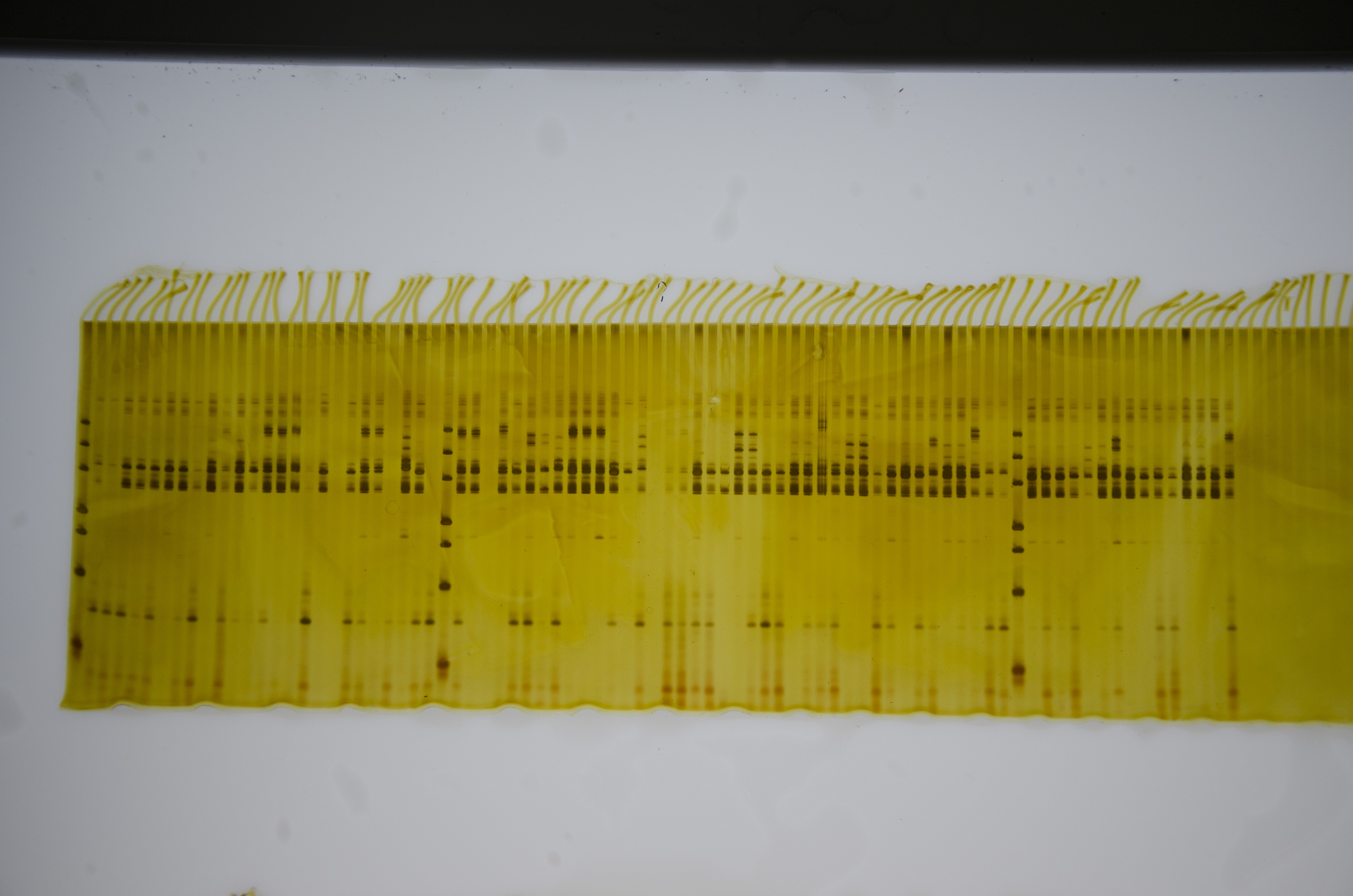

Supplement: S1 Folder — (ZIP) [file pone.0177577.s002.zip › S2 File/120.jpg]

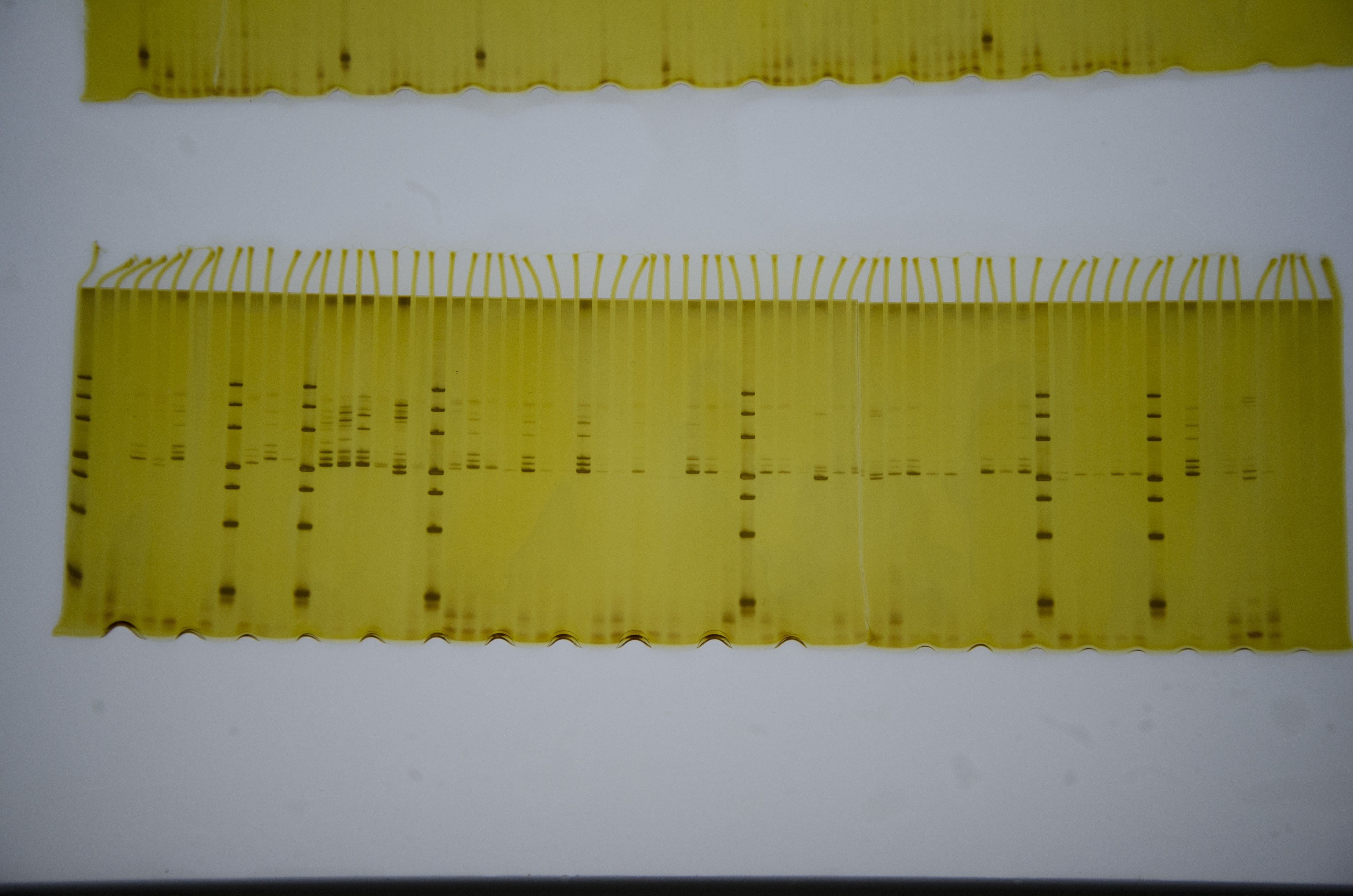

Supplement: S1 Folder — (ZIP) [file pone.0177577.s002.zip › S2 File/177.jpg]

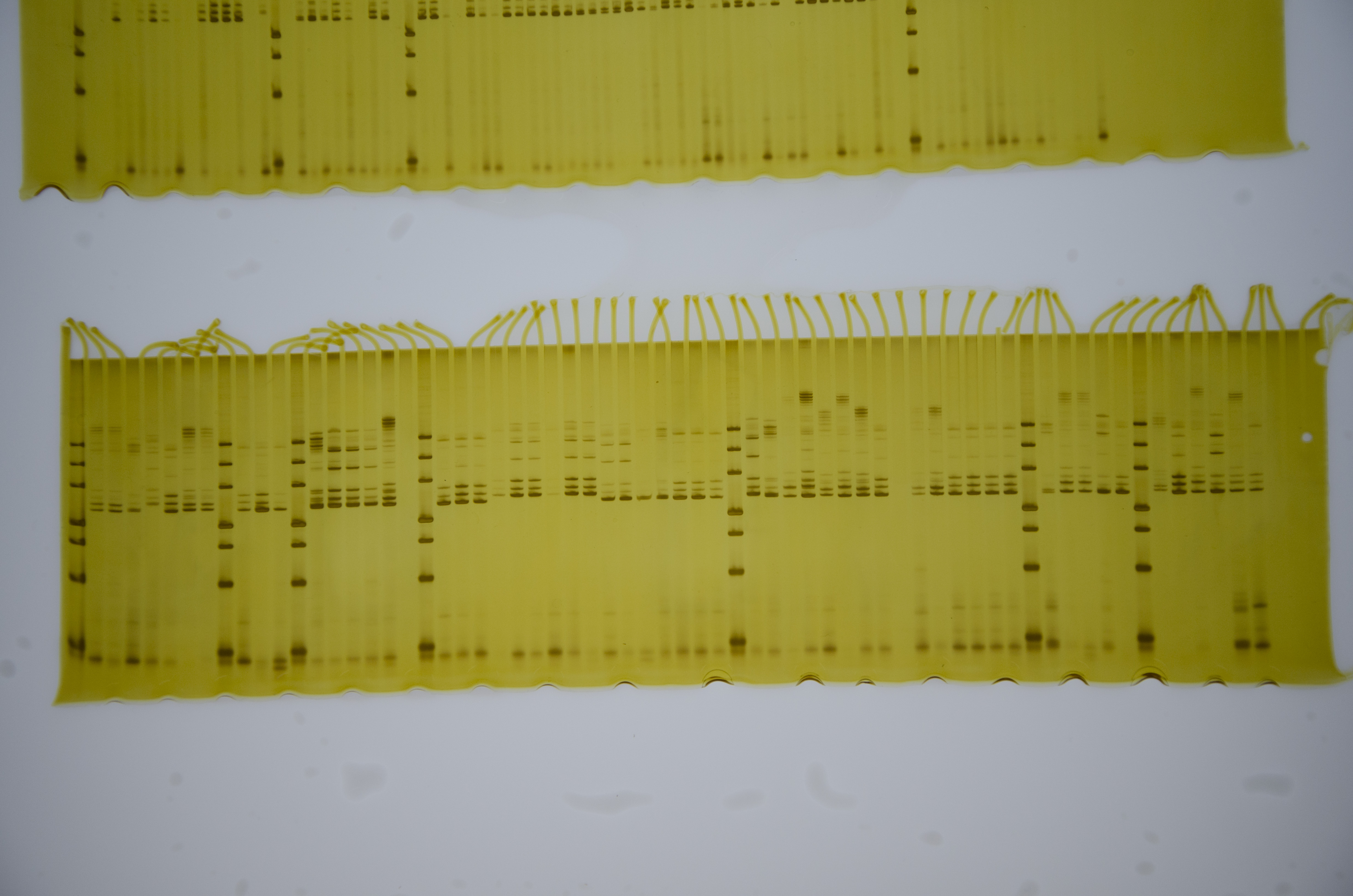

Supplement: S1 Folder — (ZIP) [file pone.0177577.s002.zip › S2 File/192.jpg]

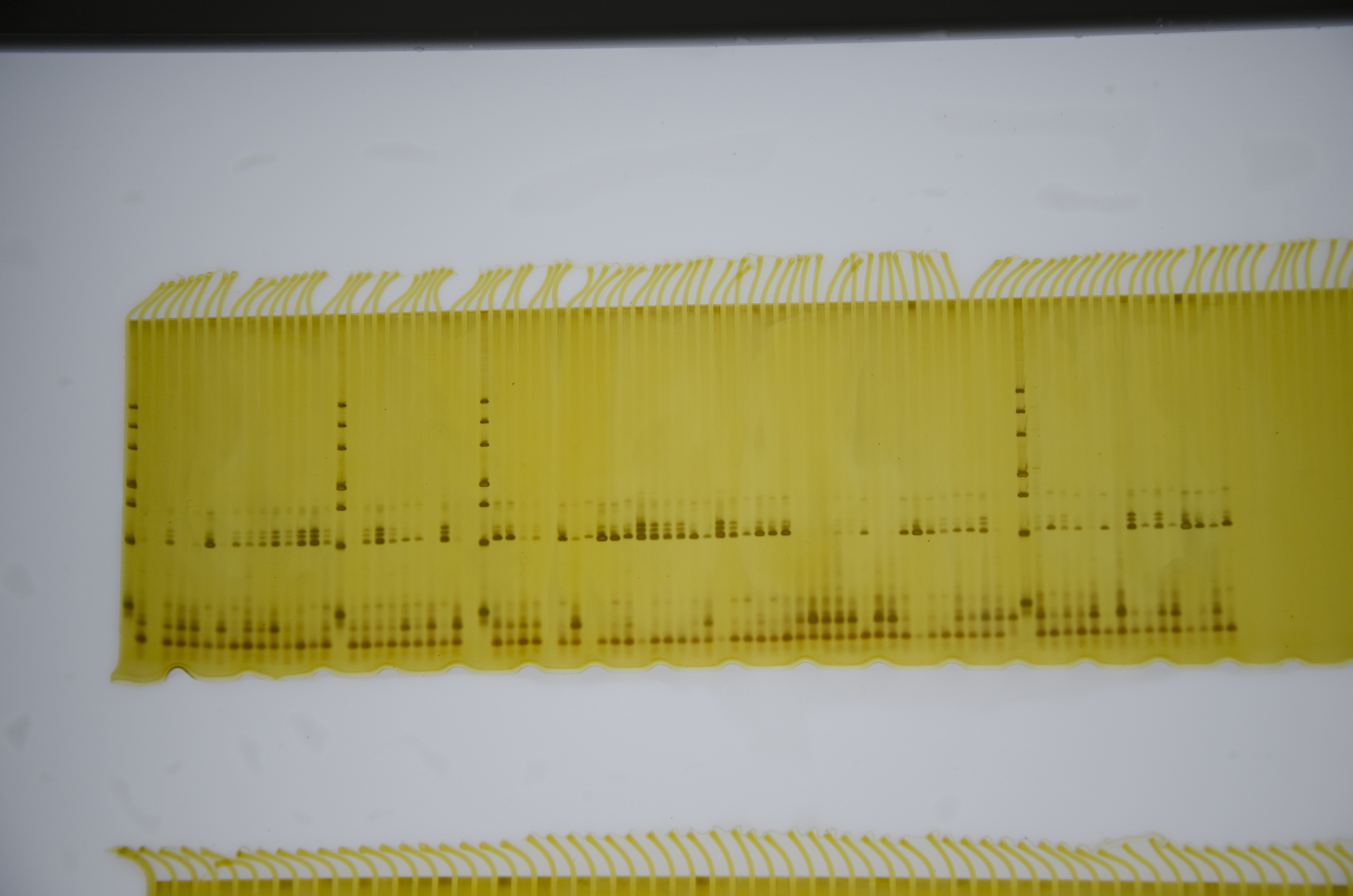

Supplement: S1 Folder — (ZIP) [file pone.0177577.s002.zip › S2 File/197.jpg]

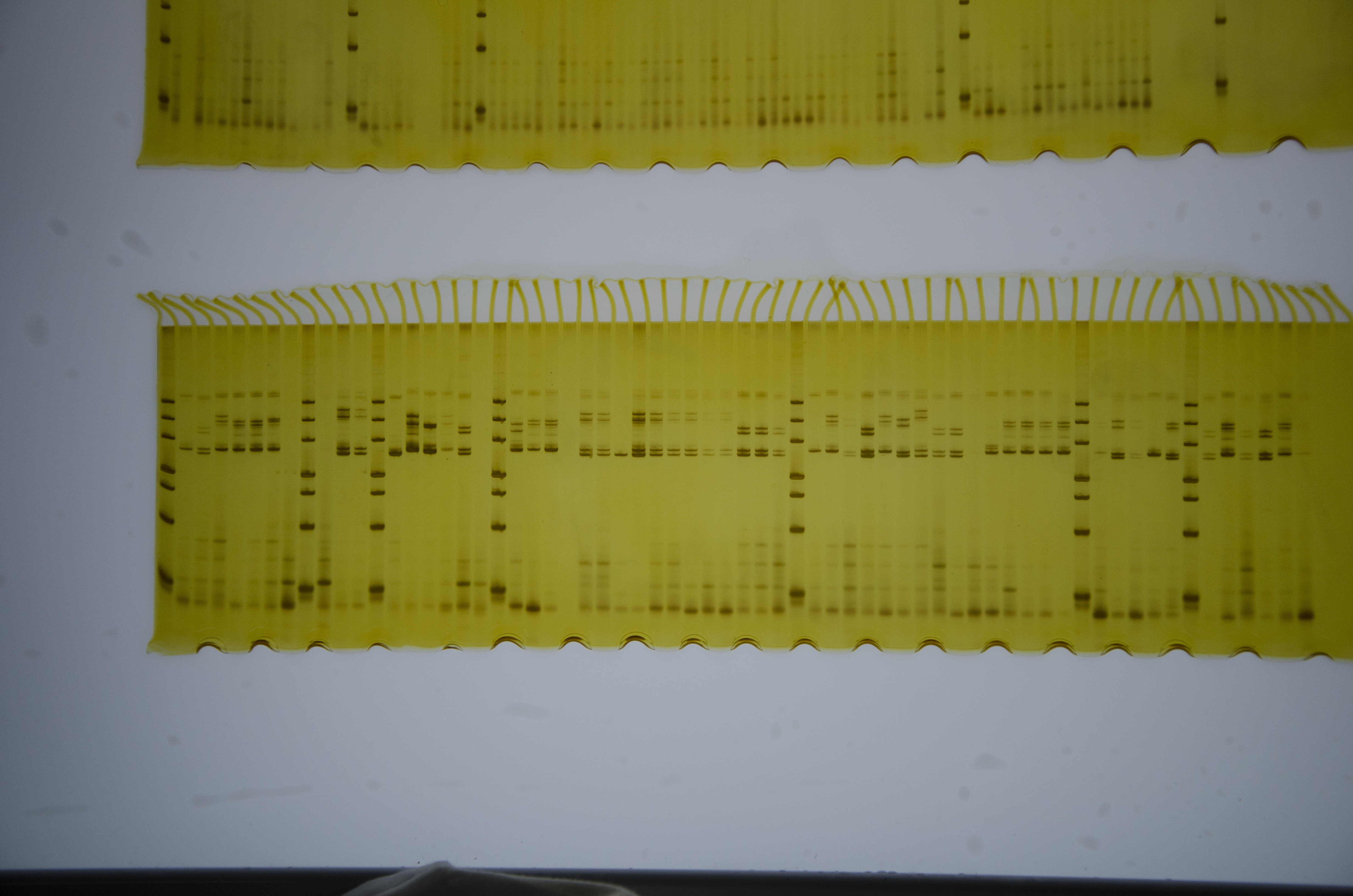

Supplement: S1 Folder — (ZIP) [file pone.0177577.s002.zip › S2 File/203.jpg]

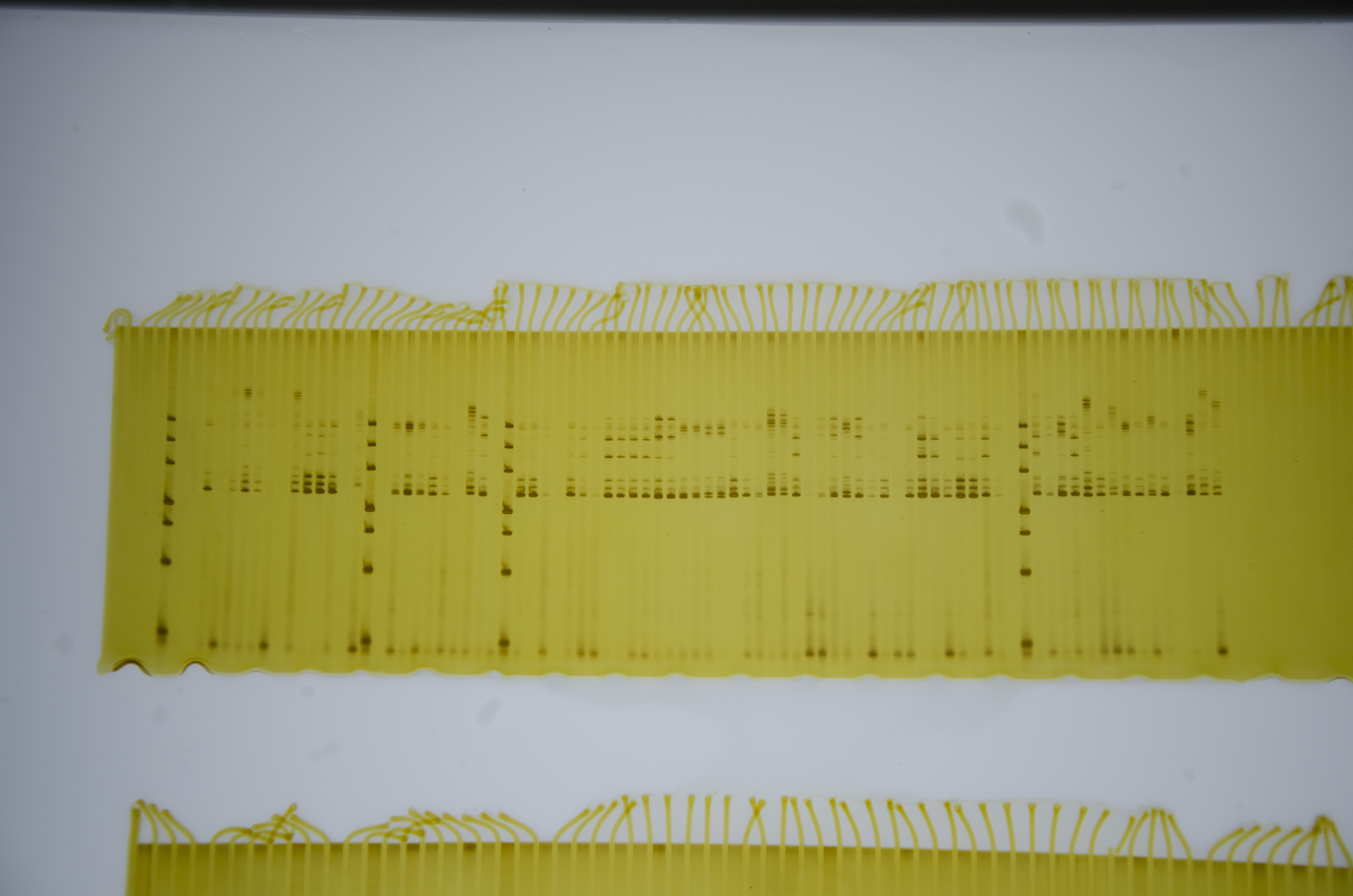

Supplement: S1 Folder — (ZIP) [file pone.0177577.s002.zip › S2 File/262.jpg]

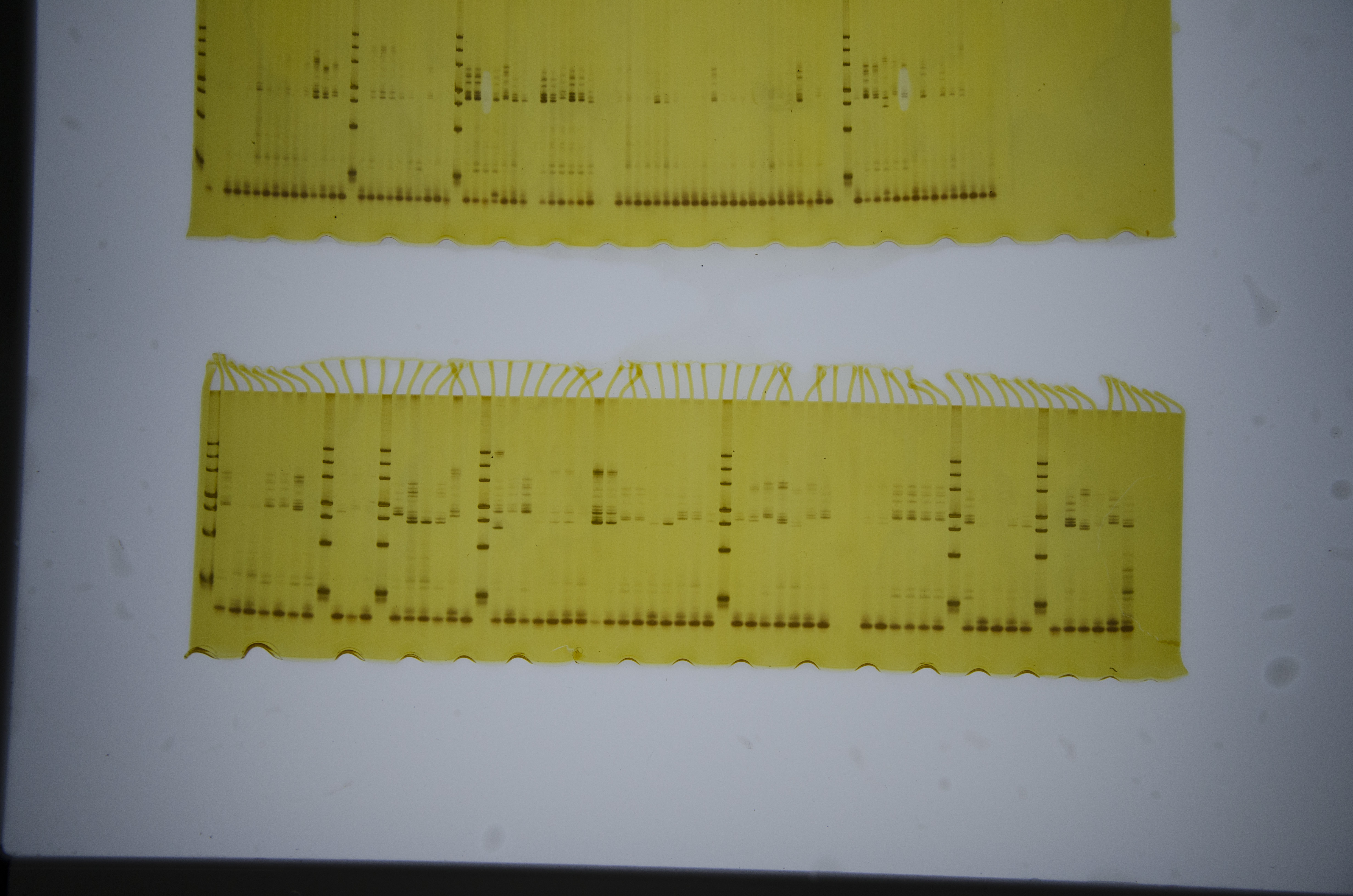

Supplement: S1 Folder — (ZIP) [file pone.0177577.s002.zip › S2 File/274.jpg]

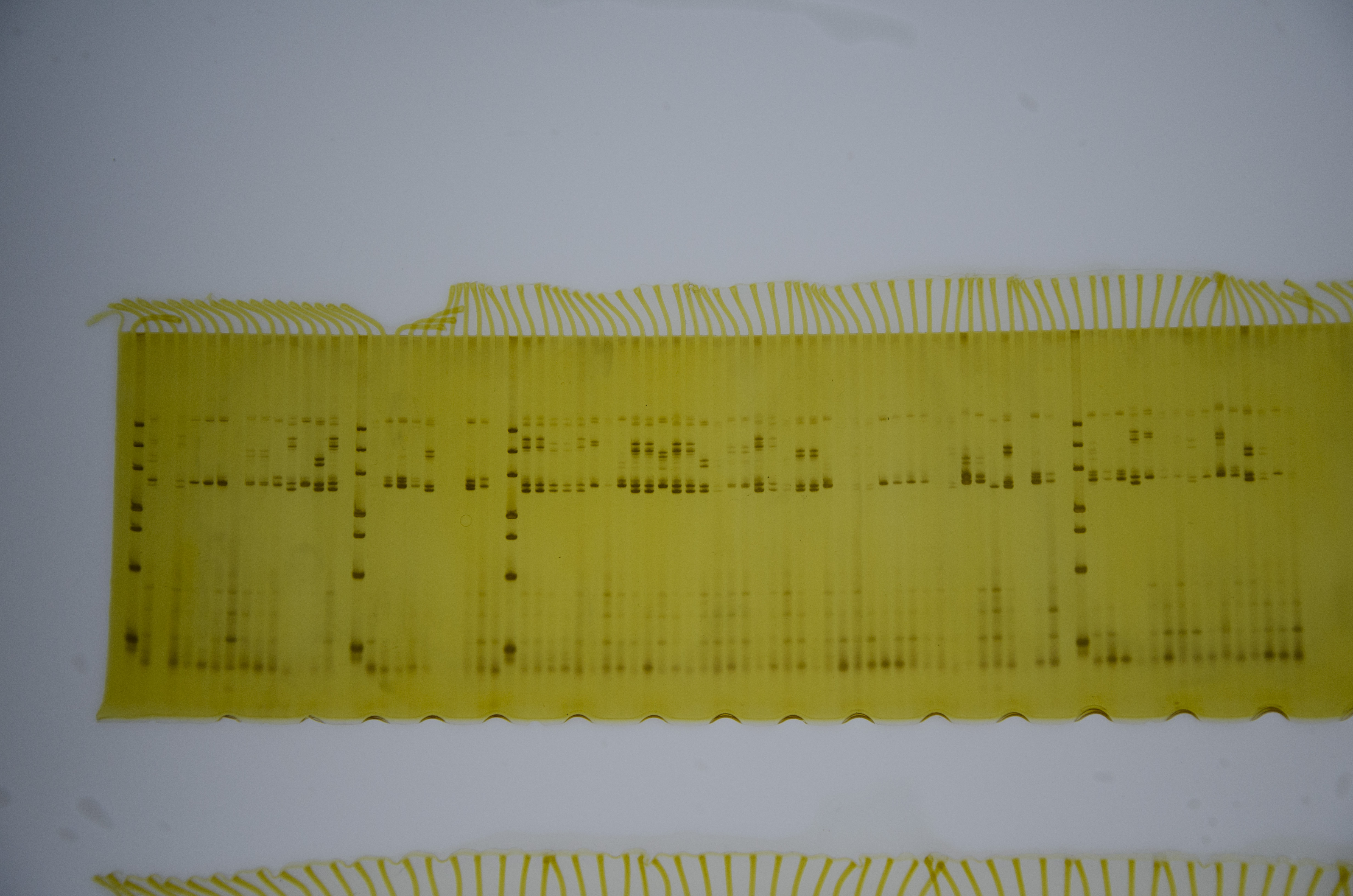

Supplement: S1 Folder — (ZIP) [file pone.0177577.s002.zip › S2 File/282.jpg]

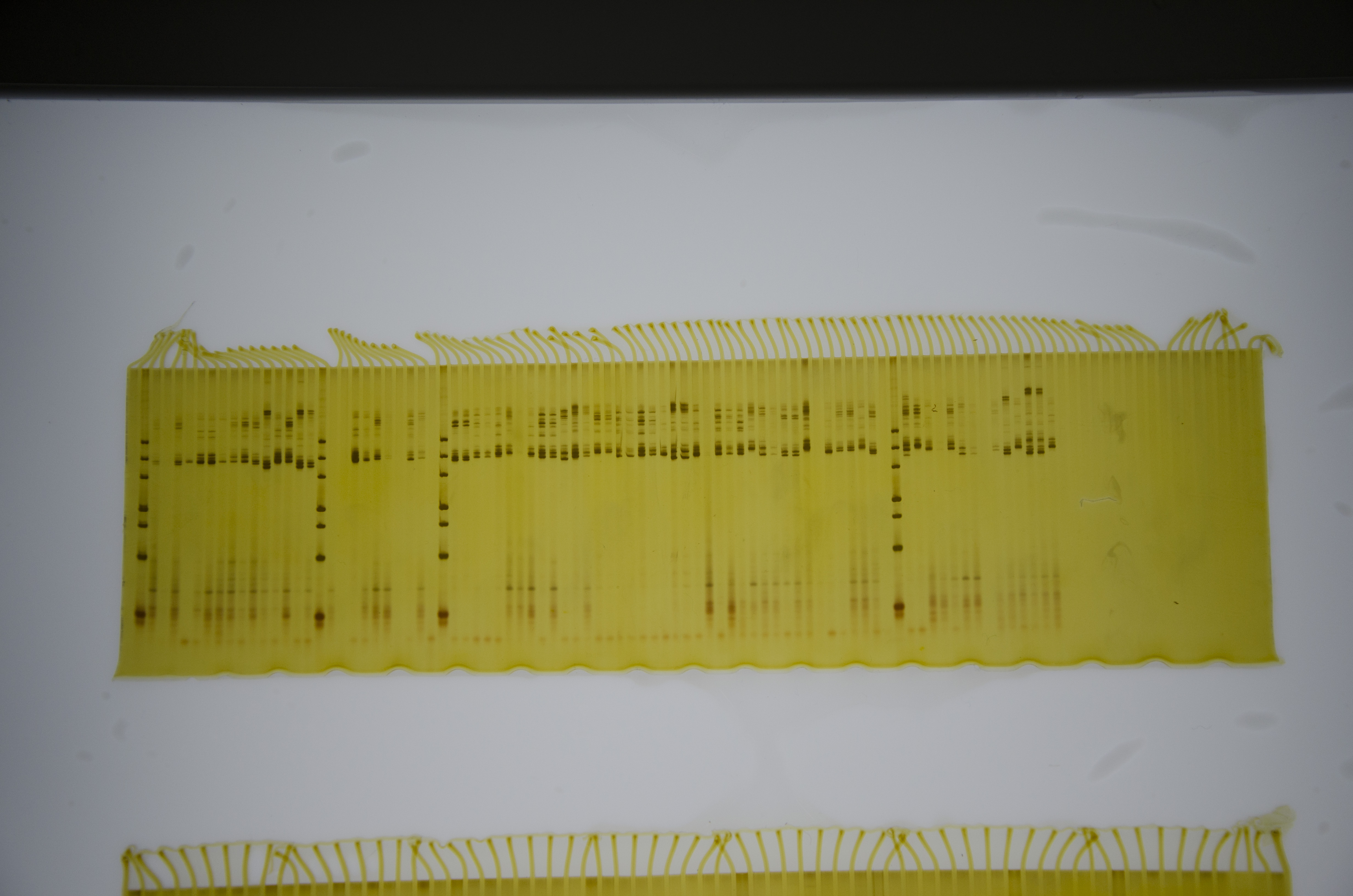

Supplement: S1 Folder — (ZIP) [file pone.0177577.s002.zip › S2 File/323.jpg]

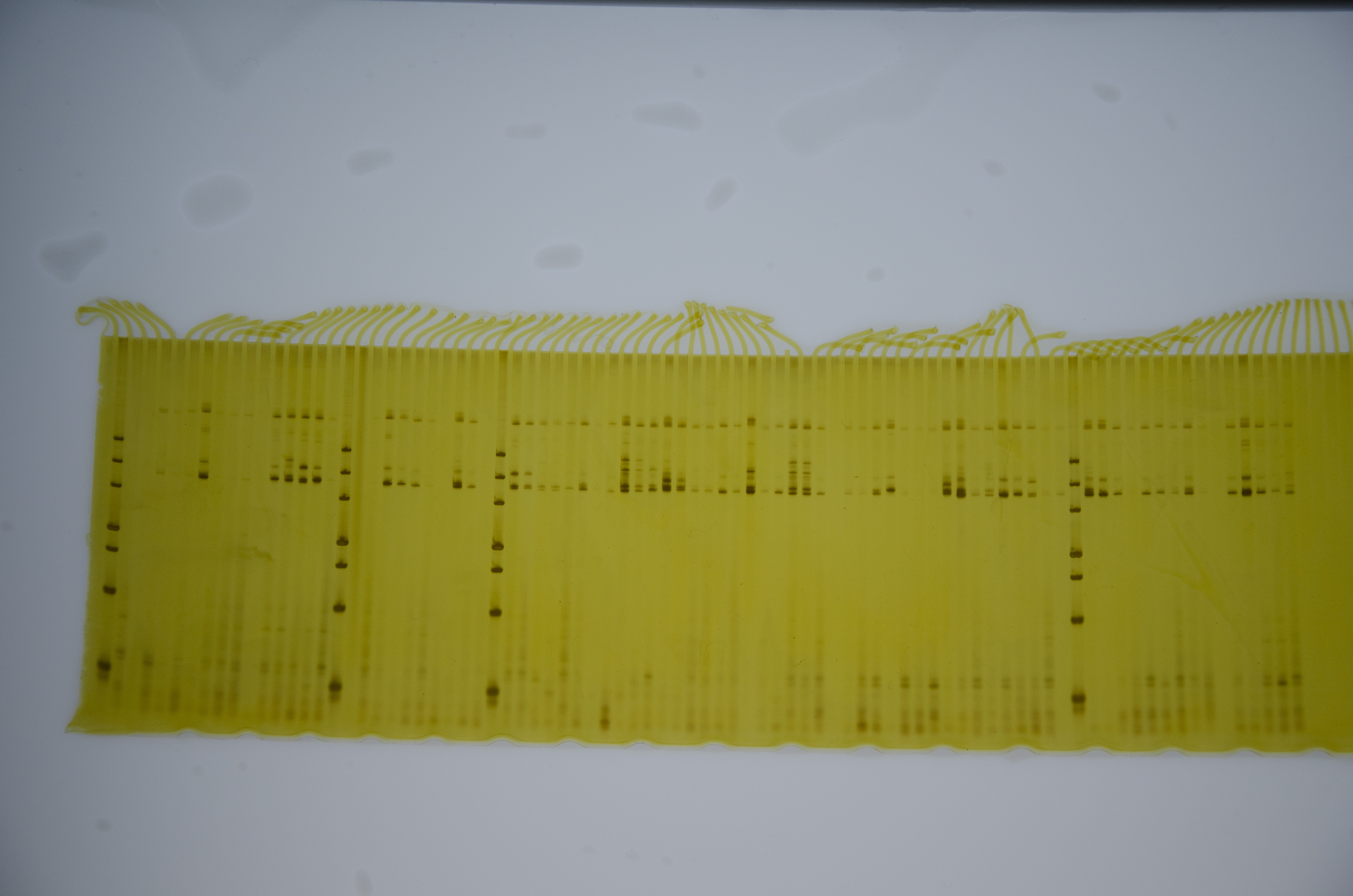

Supplement: S1 Folder — (ZIP) [file pone.0177577.s002.zip › S2 File/343.jpg]

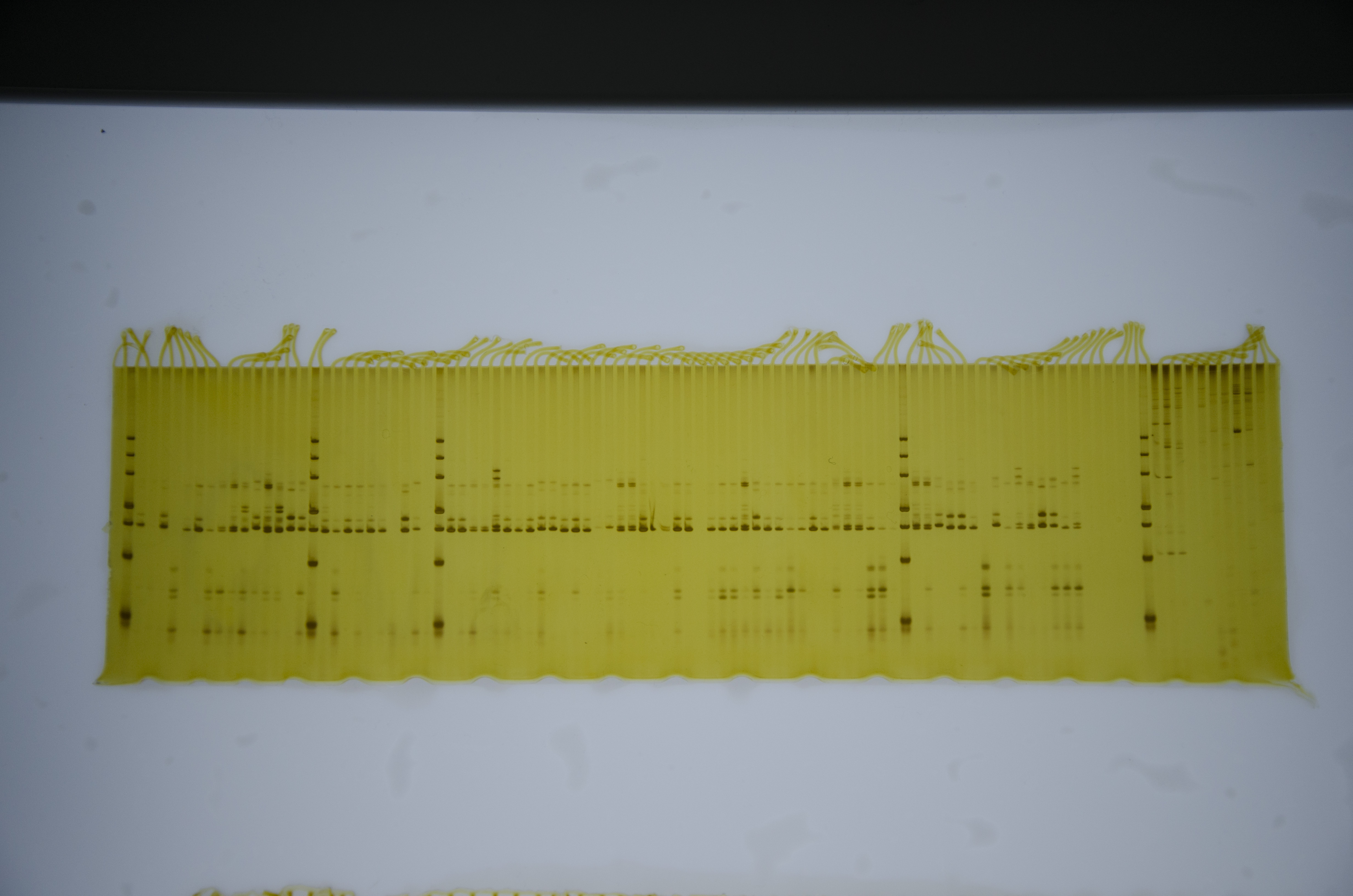

Supplement: S1 Folder — (ZIP) [file pone.0177577.s002.zip › S2 File/365.jpg]

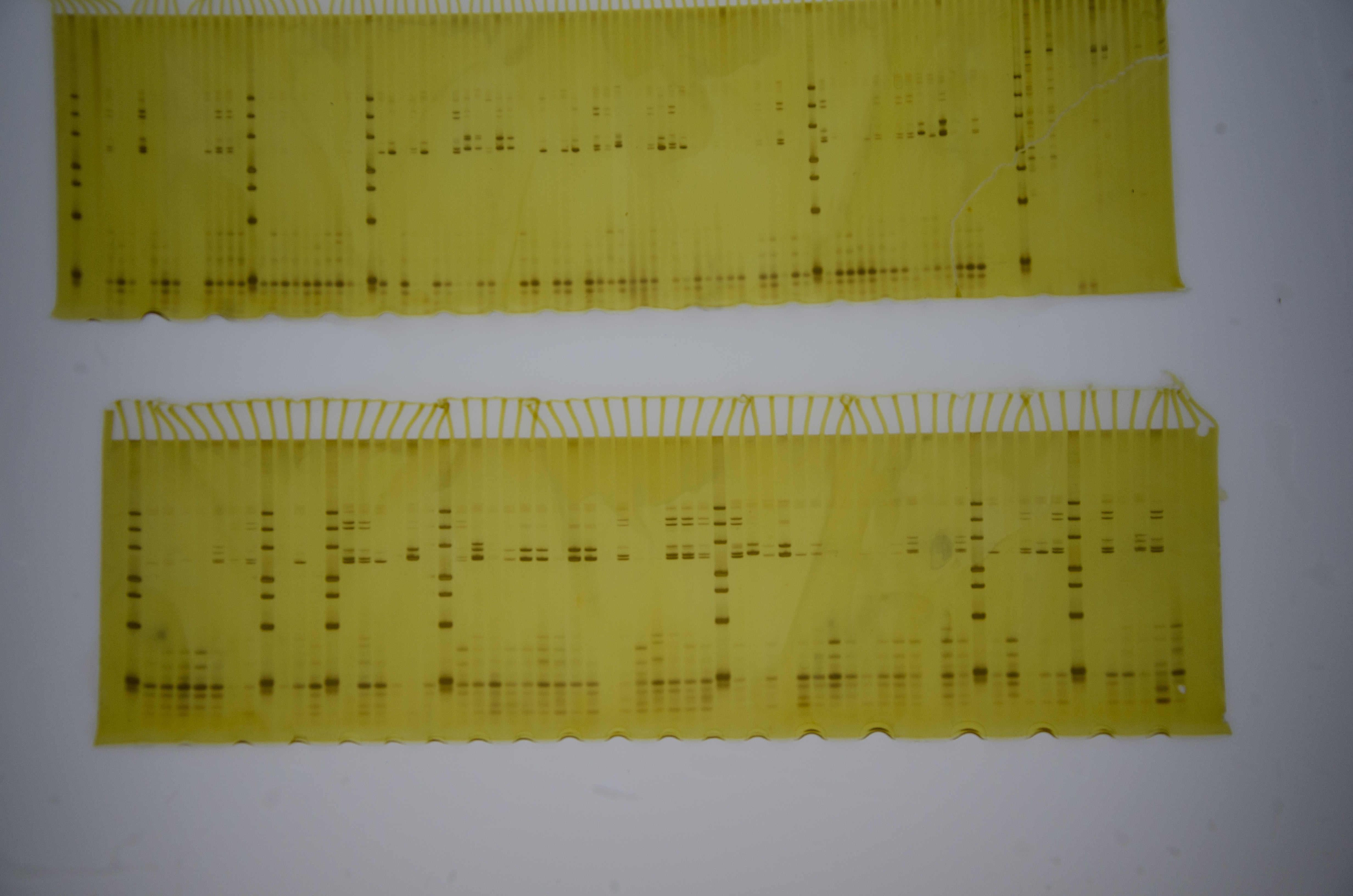

Supplement: S1 Folder — (ZIP) [file pone.0177577.s002.zip › S2 File/371.jpg]

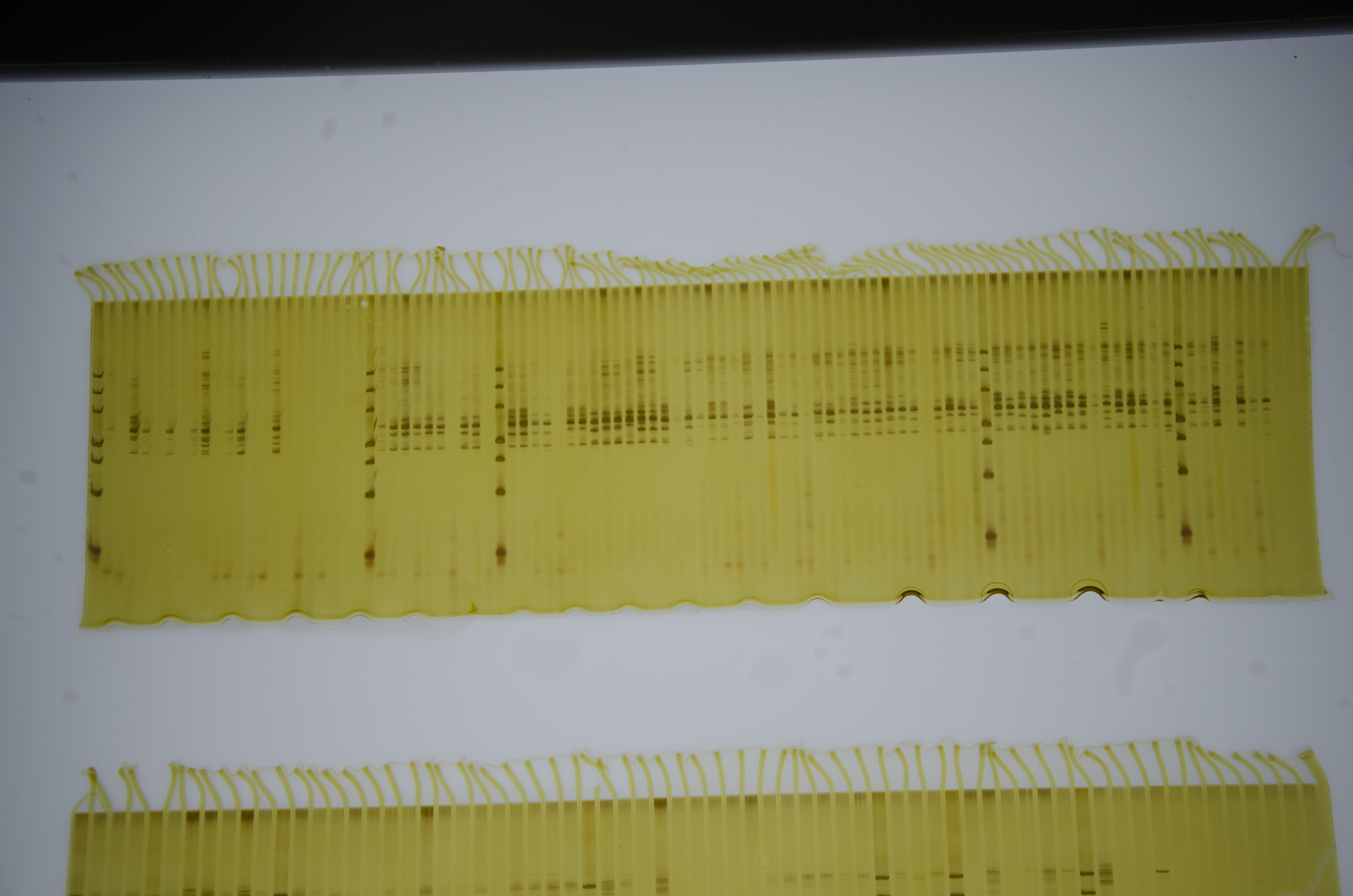

Supplement: S1 Folder — (ZIP) [file pone.0177577.s002.zip › S2 File/51.jpg]

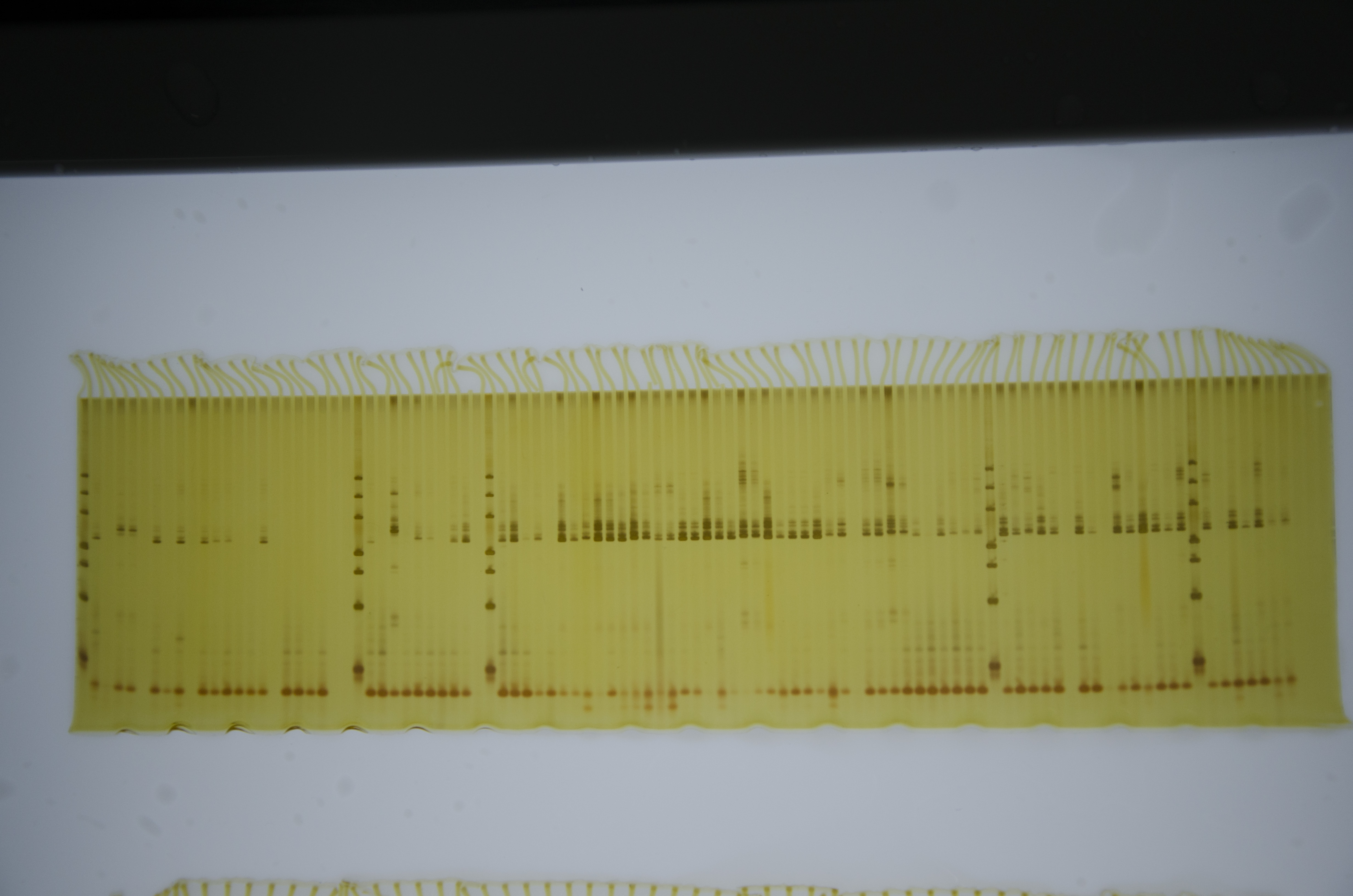

Supplement: S1 Folder — (ZIP) [file pone.0177577.s002.zip › S2 File/58.jpg]

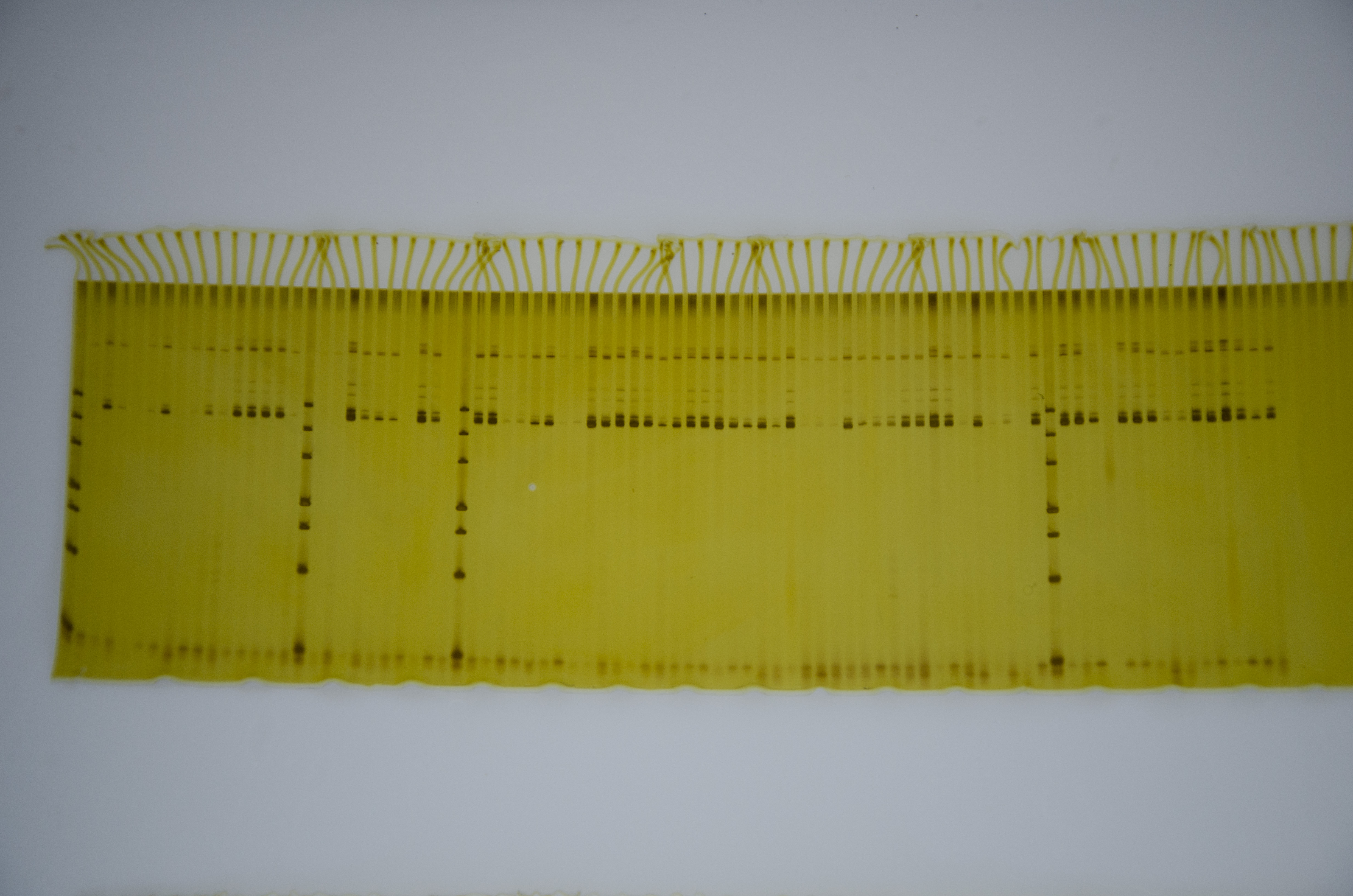

Supplement: S1 Folder — (ZIP) [file pone.0177577.s002.zip › S2 File/62.jpg]

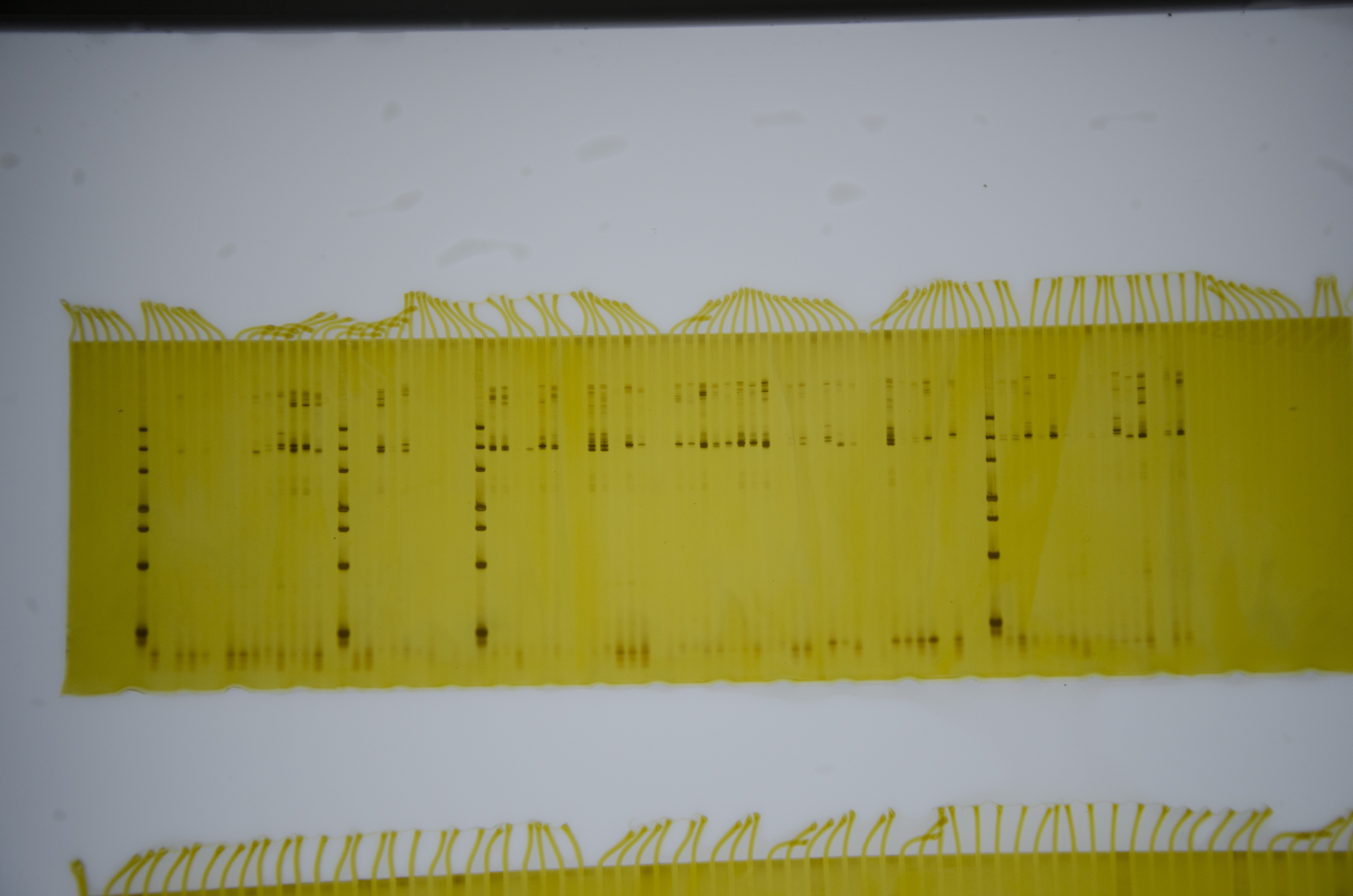

Supplement: S1 Folder — (ZIP) [file pone.0177577.s002.zip › S2 File/634.jpg]

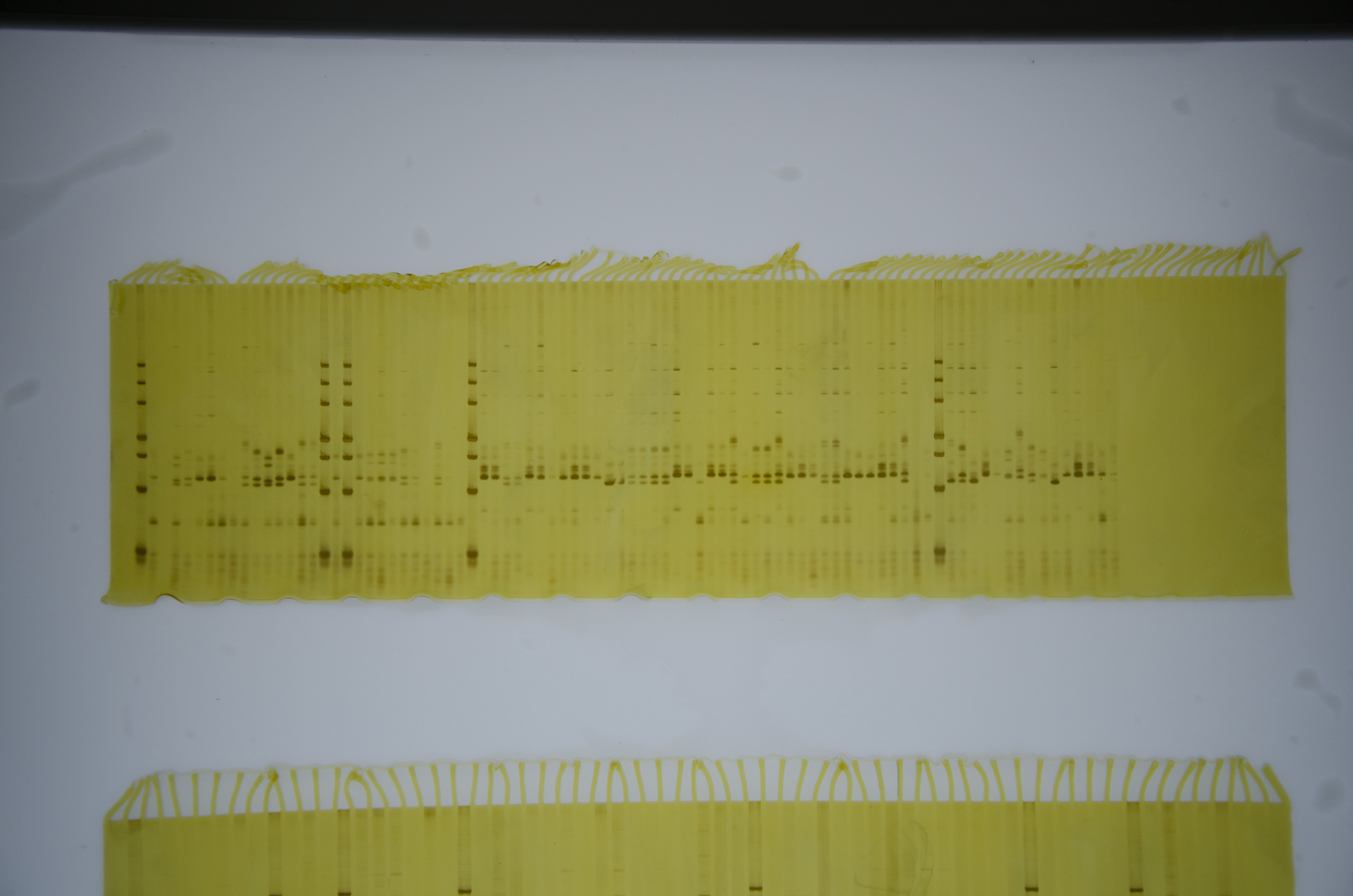

Supplement: S1 Folder — (ZIP) [file pone.0177577.s002.zip › S2 File/639.jpg]

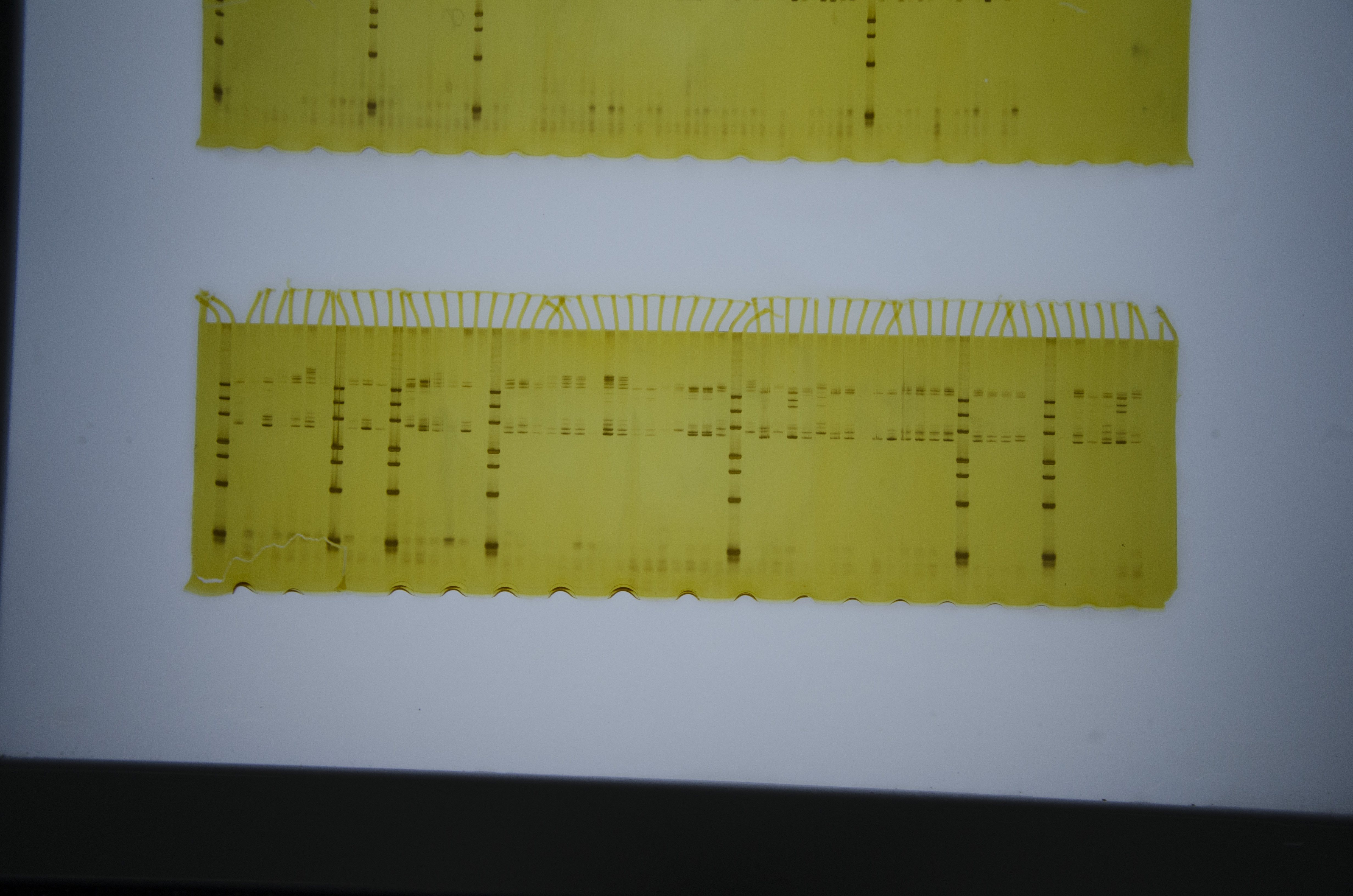

Supplement: S1 Folder — (ZIP) [file pone.0177577.s002.zip › S2 File/643.jpg]

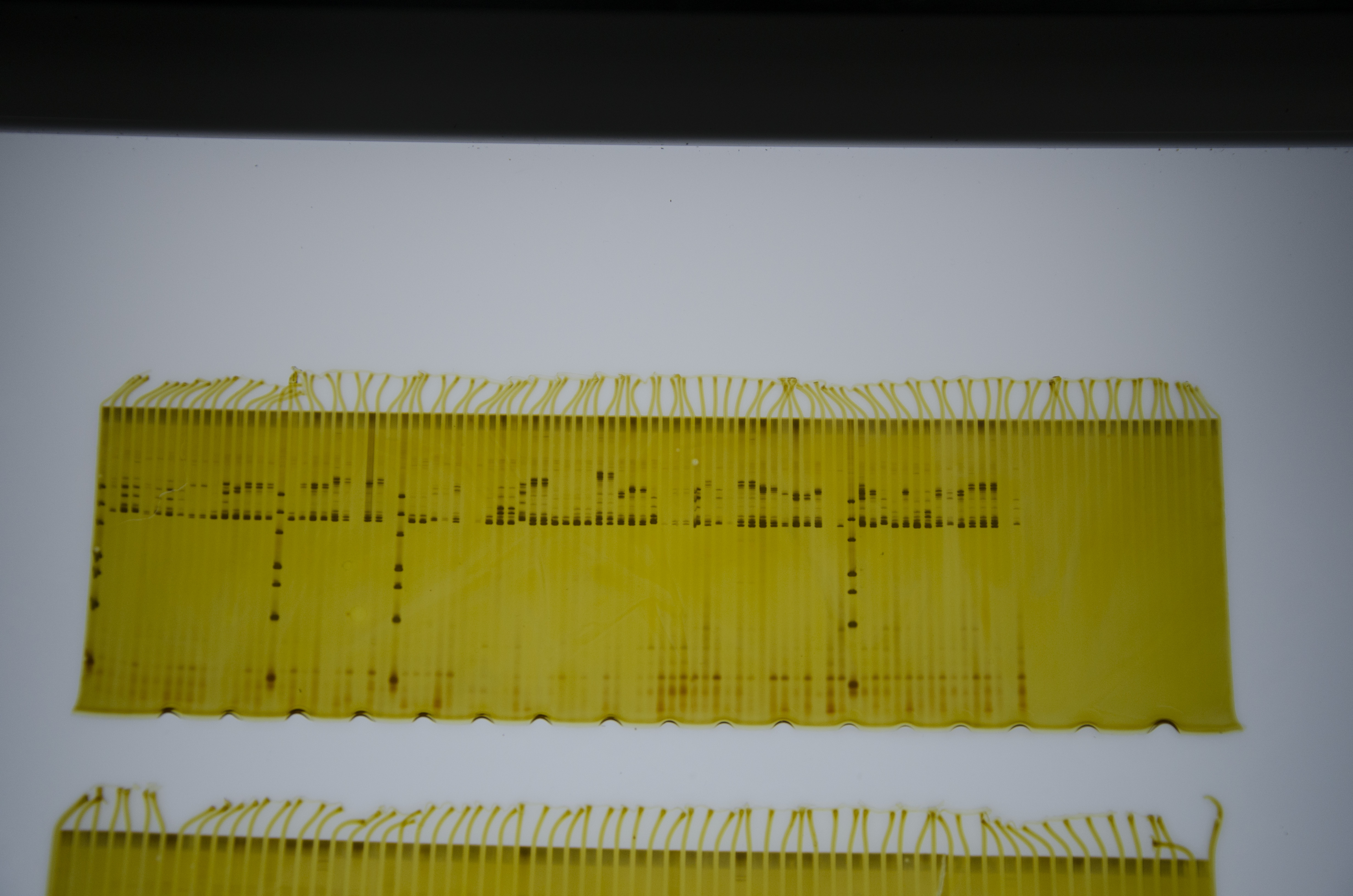

Supplement: S1 Folder — (ZIP) [file pone.0177577.s002.zip › S2 File/65.jpg]
